# Truncation of the transcriptional repressor protein Cre1 in *Trichoderma reesei* Rut-C30 turns it into an activator

**DOI:** 10.1186/s40694-018-0059-0

**Published:** 2018-08-20

**Authors:** Alice Rassinger, Agnieszka Gacek-Matthews, Joseph Strauss, Robert L. Mach, Astrid R. Mach-Aigner

**Affiliations:** 1 Institute of Chemical, Environmental and Bioscience Engineering, TU Wien, Gumpendorfer Str. 1a, 1060 Vienna, Austria; 2 Fungal Genetics and Genomics Lab, Department of Applied Genetics and Cell Biology, BOKU-University of Natural Resources and Life Sciences, Konrad Lorenz Str. 24, 3430 Tulln/Donau, Austria; 3 Institute of Microbiology, University of Veterinary Medicine Vienna, Veterinärplatz 1, 1210 Vienna, Austria

**Keywords:** Carbon catabolite repression, *Trichoderma reesei*, Cre1, Gene regulation, Transcription factor, Cellulases, Xylanases, Chromatin

## Abstract

**Background:**

The filamentous fungus *Trichoderma reesei* (*T. reesei*) is a natural producer of cellulolytic and xylanolytic enzymes and is therefore industrially used. Many industries require high amounts of enzymes, in particular cellulases. Strain improvement strategies by random mutagenesis yielded the industrial ancestor strain Rut-C30. A key property of Rut-C30 is the partial release from carbon catabolite repression caused by a truncation of the repressor Cre1 (Cre1-96). In the *T. reesei* wild-type strain a full *cre1* deletion leads to pleiotropic effects and strong growth impairment, while the truncated *cre1*-*96* enhances cellulolytic activity without the effect of growth deficiencies. However, it is still unclear which function Cre1-96 has in Rut-C30.

**Results:**

In this study, we deleted and constitutively expressed *cre1*-*96* in Rut-C30. We found that the presence of Cre1-96 in Rut-C30 is crucial for its cellulolytic and xylanolytic performance under inducing conditions. In the case of the constitutively expressed Cre1-96, the cellulase activity could further be improved approximately twofold. The deletion of *cre1*-*96* led to growth deficiencies and morphological abnormalities. An in silico domain prediction revealed that Cre1-96 has all necessary properties that a classic transactivator needs. Consequently, we investigated the cellular localization of Cre1-96 by fluorescence microscopy using an eYFP-tag. Cre1-96 is localized in the fungal nuclei under both, inducing and repressing conditions. Furthermore, chromatin immunoprecipitation revealed an enrichment of Cre1-96 in the upstream regulatory region of the main transactivator of cellulases and xylanases, Xyr1. Interestingly, transcript levels of *cre1*-*96* show the same patterns as the ones of *xyr1* under inducing conditions.

**Conclusions:**

The findings suggest that the truncation turns Cre1 into an activating regulator, which primarily exerts its role by approaching the upstream regulatory region of *xyr1*. The conversion of repressor proteins to potential activators in other biotechnologically used filamentous fungi can be applied to increase their enzyme production capacities.

**Electronic supplementary material:**

The online version of this article (10.1186/s40694-018-0059-0) contains supplementary material, which is available to authorized users.

## Background

Cellulose and hemicellulose are the most abundant biopolymers in plants. After the industrial processing of trees, crops and other plants, which are grown for food and other purposes, a lot of cellulosic and hemicellulosic waste accumulates [1]. The quality and composition of this waste can be quite versatile, depending on the branch of industry they originate from. However, they all share a significant, unused carbohydrate content that can be utilized for the production of valuable products [1]. The main challenge for an economic utilization of these waste products is the efficient conversion of cellulose-rich biomass to products such as (ligno)cellulosic ethanol [2]. One main limitation is the extraction of monomeric and dimeric sugars such as cellobiose, d-glucose and d-xylose from cellulose and hemicellulose [3]. The rigidity of the structure of cellulose and hemicellulose requires first mechanical and chemical treatment, which demand high temperatures, harsh chemicals and create an ecologically difficult disposable waste stream. Secondly, hydrolysis of cellulose and hemicellulose is done enzymatically. For the hydrolysis in industrial scale, the main bottleneck is an affordable price on bulk amounts of cellulose and hemicellulose degrading enzymes [3]. The filamentous fungus *Trichoderma reesei* (*T. reesei)* is one of the top producers of such enzymes (e.g. cellobiohydrolases (EC 3.2.1.91), endoglucanases (EC 3.2.1.4), endo-β-1,4-xylanases (EC 3.2.1.8), β-xylosidases (EC 3.2.1.37) (reviewed in [4])) in industry. Those enzymes are moderately expressed in the presence of cellulose and the hemicellulose xylan and stronger by the respective degradation products. Surprisingly, lactose also triggers the expression of these enzymes even though it is not present in the natural environment of the fungus. Although the exact induction mechanism is not fully understood, the uptake of lactose by a permease is necessary for the activation of cellulase gene expression [5].

Anyhow, the enzyme formation is limited by carbon catabolite repression (CCR) in the presence of high concentrations of easily metabolizable monomeric carbohydrates, such as d-glucose or d-xylose [6]. The uptake of d-glucose enables the fungus to rapidly gain energy; hence, the degradation of complex biopolymers by the cellulolytic and xylanolytic enzymes is shut down. The CCR mechanism is well conserved amongst various organisms ranging from bacteria to humans. Based on the sequence homologies to CreA from *Aspergillus* species, the Carbon catabolite repressor protein Cre1 (encoded by *cre1*) was described as the regulator of CCR in *T. reesei* during the 1990ies [7]. Cre1 is a C_2_H_2_ zinc finger protein and binds to a 5′-SYGGRG-3′ motif within upstream regulatory regions (URR) of cellulase and xylanase encoding genes (e.g. *cbh1* [8], *xyn1* [9]). Its regulon also comprises sugar transporters, developmental processes, and parts of the chromatin remodelling machinery such as nucleosome positioning [10, 11]. Most notably, Cre1 acts negatively on the transcription of the main and essential transactivator of cellulolytic and xylanolytic enzyme expression, Xyr1 [12]. Thus, Xyr1 is also a subject to CCR mediated by Cre1 [13]. With regards to the industry-scale production of hydrolytic enzymes, top producing *T. reesei* strains became a necessity. Random mutagenesis yielded the mutant strain Rut-C30, which achieves enzyme yields of 20 g/L [14]. The nowadays used industrial *T. reesei* strains (yielding up to 100 g/L [15]) are based on Rut-C30 and thus share a similar genetic background. Predominately, this includes a truncation of Cre1, which led to partial de-repression from CCR on d-glucose [16]. Nevertheless, with regards to the wild-type system, we refer in this manuscript to d-glucose as a repressing condition. In 2014, Mello-de-Sousa and colleagues used the *T. reesei* wild-type strain to demonstrate that this truncated Cre1 (Cre1-96) positively influences cellulase expression, while the full deletion of *cre1* leads to strong pleiotropic effects and growth impairment [17]. The enhancement of cellulase expression by Cre1-96 was attributed to a chromatin opening in the URR of cellulase-encoding genes and also of the *xyr1* gene. However, the impact of Cre1-96 was never studied directly in Rut-C30. The exact regulatory mechanism of Cre1-96 and its role as a putative new transcription factors in industrial strains still remain to be elucidated. In this study, we investigated the effects of a *cre1*-*96* deletion in Rut-C30 on its growth behaviour, the enzymatic activities and the transcriptional profiles of cellulase- and xylanase-encoding genes (*cbh1*, *xyn1*) and of *xyr1*. To determine the subcellular localization of the putative transcription factor, the nuclear import was examined under cellulase inducing and repressing conditions. Moreover, we performed chromatin immunoprecipitation and nuclease digestion to learn which genes are targeted by Cre1-96 and what is its impact on the DNA accessibility within the URR of its target genes. Finally, we constitutively expressed *cre1*-*96* in Rut-C30 and examined the impact on the cellulolytic activities.

## Results

### Deletion and constitutive expression of *cre1*-*96* in *T. reesei* Rut-C30

To identify the function of Cre1-96 in Rut-C30, the encoding gene was deleted from the genome. Therefore, a deletion cassette was integrated by homologous recombination at the *cre1*-*96* locus, resulting in a gene replacement of *cre1*-*96* in Rut-C30. Two *cre1*-*96* deletion strains were identified by diagnostic PCR (Additional file 1: Figure S1). Both deletion strains were used throughout this study and are in the following termed Rut-C30Δ*cre1*-*96* (1) and (2) in the figures. In the parent strain Rut-C30, the structural gene of *cre1*-*96* was put under the control of the *tef1* promoter. The homologous integration of this expression cassette at the *cre1*-*96* locus was again verified by diagnostic PCR and the resulting strain is in the following termed Rut-C30OE*cre1*-*96* (Additional file 2: Figure S2).

### Cre1-96 is required for cellulolytic and xylanolytic performance of Rut-C30

**Fig. 1:**
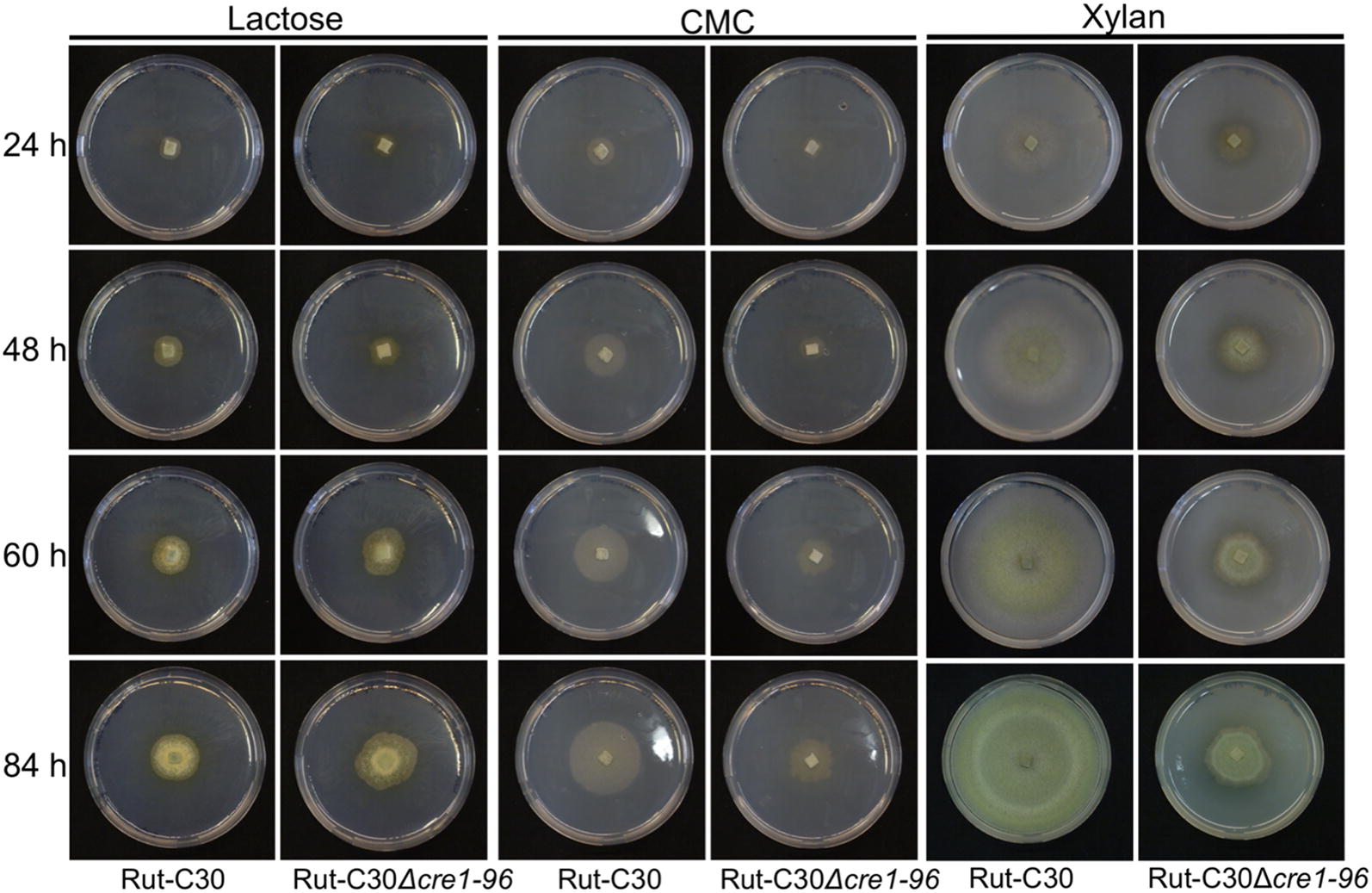
Growth behaviour of Rut-C30Δ*cre1*-*96* under cellulase inducing conditions. The *T. reesei* strains Rut-C30 and Rut-C30Δ*cre1*-*96* were pre-grown on MEX plates and were then transferred in biological duplicates to MA medium plates supplemented with 1% (w/v) lactose, CMC or xylan. Plates were incubated at 30 °C and pictures were taken after 24, 48, 60 and 84 h

To investigate a possible impact of Cre1-96 on cellulase and xylanase gene expression, the *cre1*-*96* deletion strain and its parent strain Rut-C30 were grown on plates containing lactose or carboxymethylcellulose (CMC) to resemble cellulase-inducing conditions (Fig. 1). Further, they were grown on xylan for induction of xylanase expression (Fig. 1), on a non-inducing condition (glycerol) and on a repressing condition (d-glucose) (Additional file 3: Figure S3). Photos of the plates were taken after 24, 48, 60 and 84 h of growth. On lactose, no clear differences in growth were obtained between the two tested strains at any time point (Fig. 1). However, the radial colony formation seemed to be abnormal after 60 and 84 h when *cre1*-*96* was absent (Fig. 1). On CMC and xylan, growth deficiencies were observed in the *cre1*-*96* deletion strain at all times points in comparison to the parent strain. The colony was clearly reduced in size, while no influence on sporulation was visible (Fig. 1). On glycerol and d-glucose no obvious growth reduction was visible at any time point. However, the spore pigmentation changed in colour intensity (from yellow to light yellow or white) on glycerol and in shade (from green or yellow to brownish) on d-glucose comparing the *cre1*-*96* deletion strain to Rut-C30 after 60 and 84 h (Additional file 3: Figure S3).

**Fig. 2:**
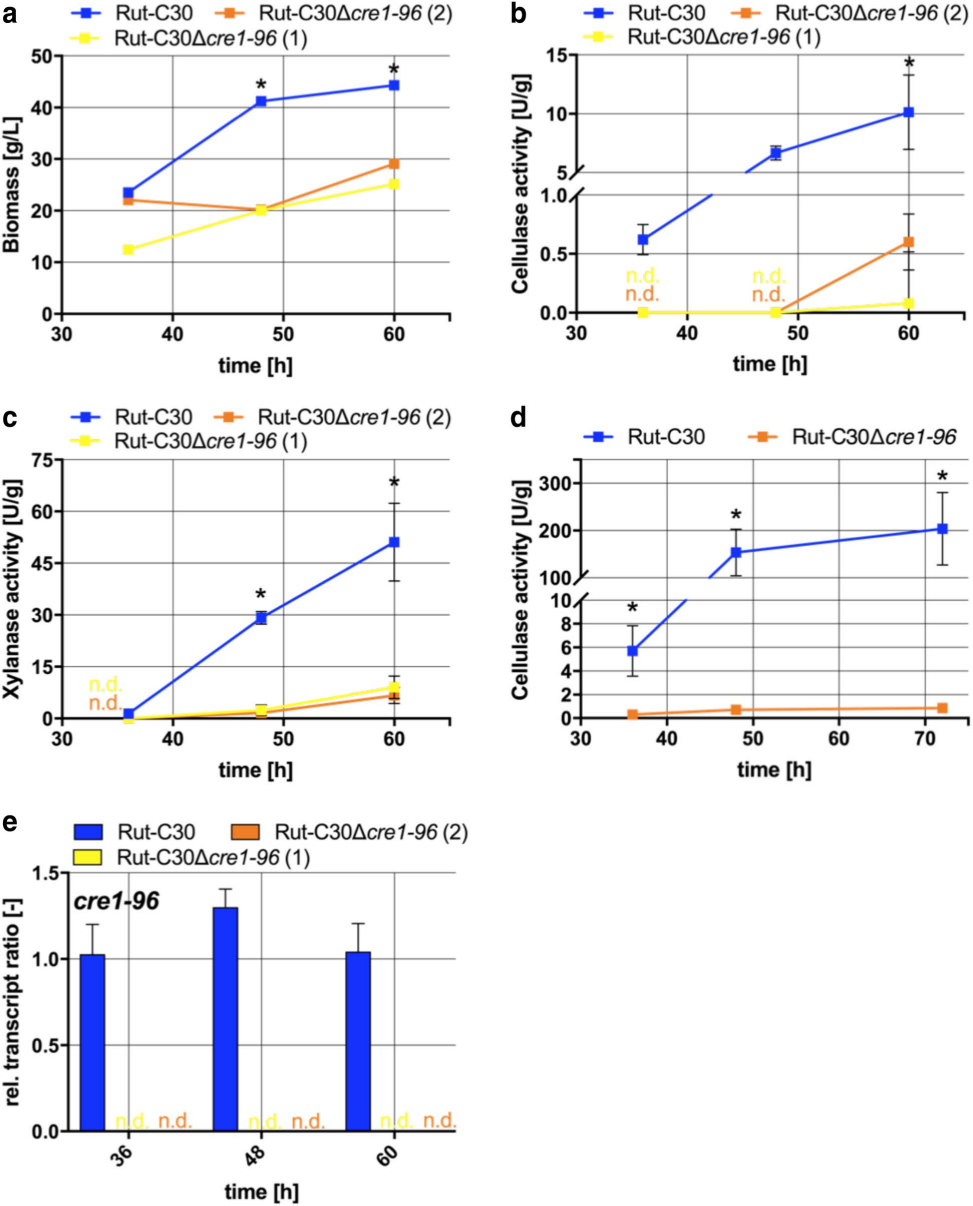
Cellulolytic and xylanolytic activities in absence and presence of Cre1-96*. T. reesei* strains Rut-C30 (blue squares) and both Rut-C30Δ*cre1*-*96* strains (yellow and orange squares) were cultivated in liquid medium supplemented with 1% (w/v) lactose or d-glucose for 36, 48 and 60 h. The endo-cellulolytic on lactose (**b**) and on glucose (**d**) as well as the xylanolytic activities on lactose (**c**) in the culture supernatants were measured in biological and technical duplicates and normalized to the biomass measured as wet weight (**a**). The enzymatic activities are given as means and the error bars indicate the standard deviations. The values were statistically analysed by an unpaired two-tailed *t* test in a confidence interval of 95%, and asterisks indicate significant differences. **e** Relative *cre1*-*96* transcript ratios were analysed for both deletion strains and Rut-C30 grown on lactose. Transcript analysis was performed in biological and technical duplicates by qPCR, data were normalized to the housekeeping genes *sar1* and *act,* and referred to the transcript level of Rut-C30 at 36 h. The relative transcript ratios are given as means and the error bars indicate the standard deviations. Error bars are not shown for standard deviations ≤ 3.5%. All values were statistically analysed in a confidence interval of 95%; ‘n.d.’ means not detected

To learn whether the slower growth of the strains carrying the *cre1*-*96* deletion results from less cellulase and xylanase activity, we tested supernatants from cultivations under inducing conditions (lactose) but also under repressing conditions by enzymatic assays. Supplementary, the abundance of *cre1*-*96* transcript was determined under inducing conditions. In contrast to the growth experiments on plates, the biomass formation in the liquid cultures was now also reduced on lactose in the Δ*cre1*-*96* strains (Fig. 2a). For this reason, the obtained cellulolytic and xylanolytic activities (Fig. 2b, c) were normalized to the biomass. Normalized to the biomass, the *cre1*-*96* deletion caused a complete loss of cellulolytic and of xylanolytic activity at earlier time points (36 and 48 h) and a strong reduction is observed after 60 h (Fig. 2b, c). Expression of *cre1*-*96* itself was equally high at all time points under inducing conditions and necessary for the enzyme production (Fig. 2e). Obviously, the presence of *cre1*-*96* is needed for a good performance in cellulase and xylanase production. Importantly, cellulolytic activities were also lost when d-glucose is used as the carbon source, which is not the case in the parent strain Rut-C30 (Fig. 2d). This reflects that in Rut-C30 the production of cellulases and xylanases is positively influenced by the presence of Cre1-96 regardless if inducing or repressing conditions are prevailing.

### Cre1-96 influences the transcript formation of *cbh1*, *xyn1* and *xyr1*

**Fig. 3:**
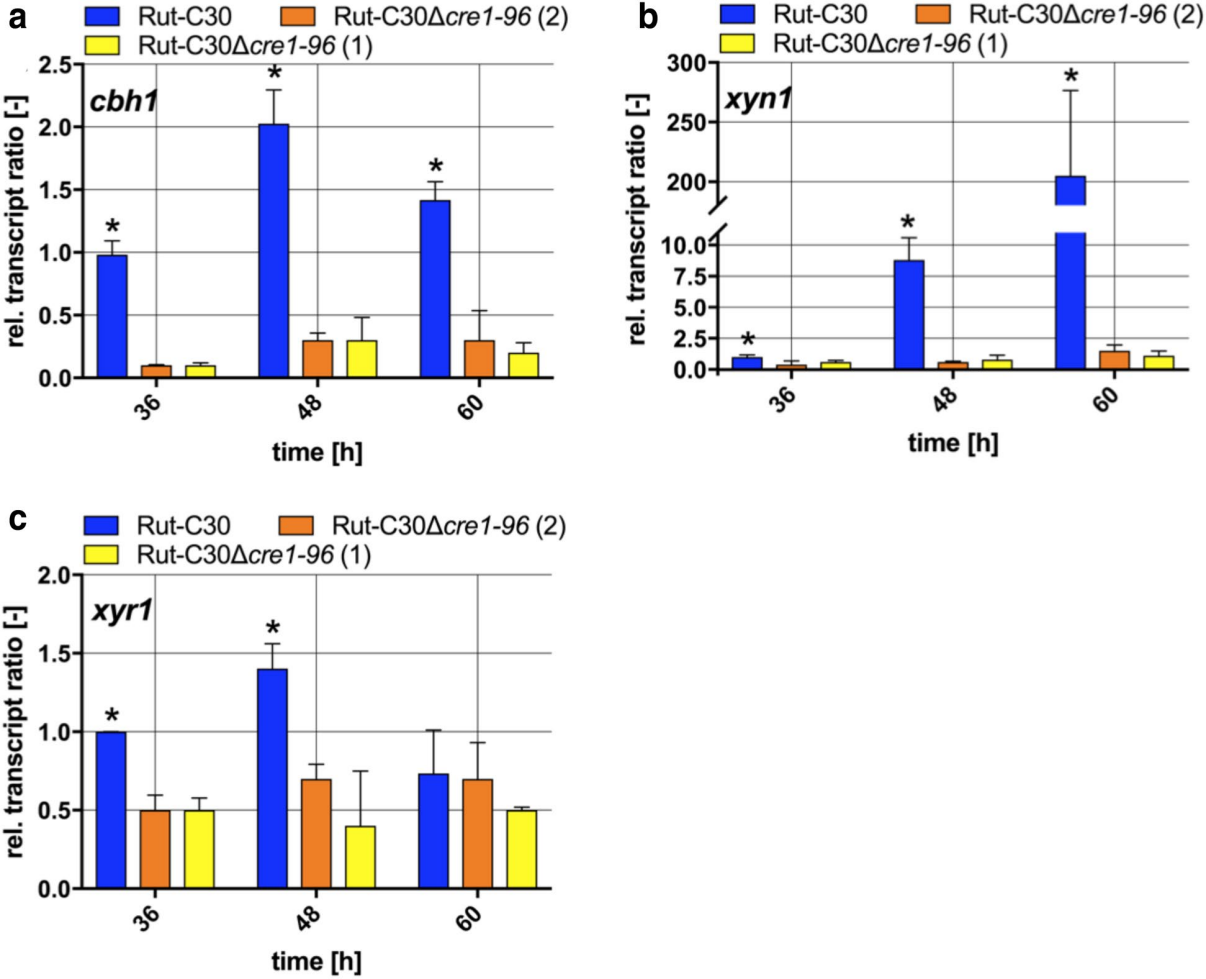
Transcript levels of *cbh1*, *xyn1* and *xyr1* in absence and presence of Cre1-96. *T. reesei* strains Rut-C30 (blue bars) and both Rut-C30Δ*cre1*-*96* strains (yellow and orange bars) were cultivated in liquid medium supplemented with 1% (w/v) lactose for 36, 48 and 60 h. Transcript analyses of *cbh1* (**a**), *xyn1* (**b**) and *xyr1* (**c **) were performed in biological and technical duplicates by qPCR, data were normalized to the housekeeping genes *sar1* and *act,* and referred to the respective transcript levels of Rut-C30 at 36 h. The relative transcript ratios are given as means and the error bars indicate the standard deviations. All values were statistically analysed in a confidence interval of 95% and asterisks indicate significant differences

The findings on the reduced enzyme activities prompted us to examine whether Cre1-96 regulates Cre1-target genes on the transcriptional level under inducing conditions. Therefore, we measured the transcript levels of *cbh1*, *xyn1* and *xyr1* on lactose in Rut-C30 and both *cre1*-*96* deletion strains. In the case of *cbh1*, the transcript levels were significantly reduced in the deletion strains compared to the parent strain at all time points (Fig. 3a). In the case of *xyn1*, the transcript levels were also significantly reduced in the deletion strains compared to its parent strain (Fig. 3b). Generally spoken, the *cbh1* and *xyn1* transcriptional profiles matched the measured enzymatic activities in Rut-C30 and both deletion strains. Interestingly, the transcript levels of *xyr1* were also reduced in the deletion strains compared to its parent strains after 36 and 48 h (Fig. 3c), but not any more at the later time point (60 h). To summarize, Cre1-96 has an impact on the formation of *cbh1* and *xyn1* transcript levels, and also on those of the main activator Xyr1 under inducing conditions.

### Cre1-96 only indirectly regulates genes involved in the lactose metabolism

**Fig. 4:**
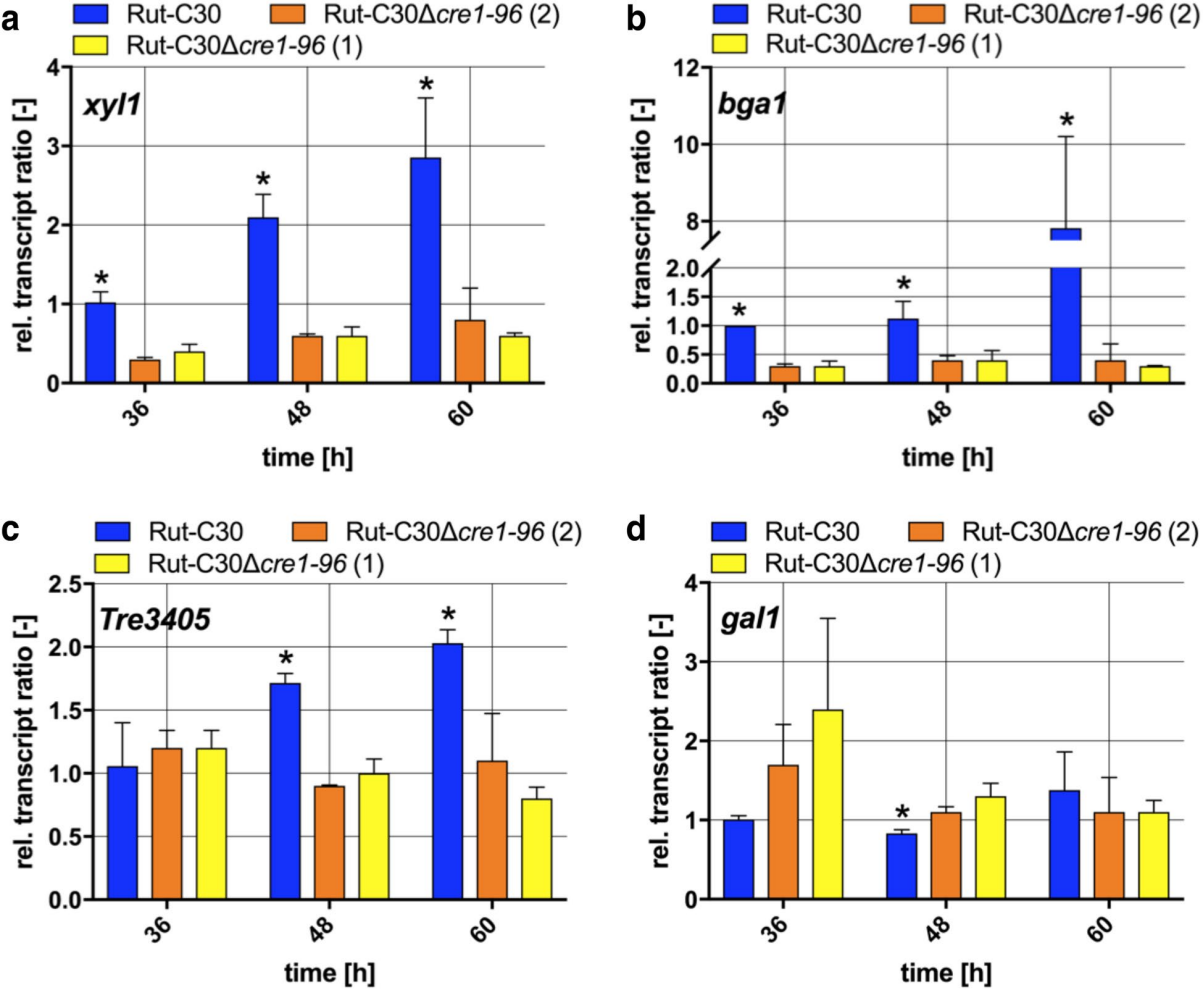
Transcript levels of *xyl1*, *bga1* and *Tre3405* in absence and presence of Cre1-96*. T. reesei* strains Rut-C30 (blue bars) and both Rut-C30Δ*cre1*-*96* strains (yellow and orange bars) were cultivated in liquid medium supplemented with 1% (w/v) lactose for 36, 48 and 60 h. Transcript analyses of *xyl1* (**a**), *bga1* (**b**) and *Tre3405* (**c**) were performed in biological and technical duplicates by qPCR, data were normalized to the housekeeping genes *sar1* and *act,* and referred to the respective transcript levels of Rut-C30 at 36 h. The relative transcript ratios are given as means and the error bars indicate the standard deviations. All values were statistically analysed in a confidence interval of 95% and asterisks indicate significant differences

The observed differences in the biomass formation in liquid culture on lactose (compare Fig. 2a) gave rise to the possibility that the lactose metabolism could be altered in the *cre1*-*96* deletion strains. Several genes are necessary for the conversion of lactose to D-galactose and d-glucose. The lactose hydrolysis depends on the extracellular β-galactosidase Bga1 and on the d-xylose reductase Xyl1. A deletion of *xyl1* results in reduced growth on lactose, which is explained by low transcript levels of *bga1* [18]. Here, we investigated the genes coding for the d-xylose reductase (*xyl1*), the β-galactosidase (*bga1*), the galactokinase (*gal1*) and a lactose specific permease (*Tre3405*) [5]. A previous study demonstrated that Xyr1 is involved in the regulation of some lactose metabolism genes by activating *xyl1* and *bga1,* but not *gal1* transcription [19]. As it seems that Cre1-96 has an influence on the *xyr1* transcript formation (compare Fig. 3c), it is very likely that also genes involved in the lactose metabolism are affected by the *cre1*-*96* deletion. Significantly reduced transcripts of the *xyl1*, *bga1* and *Tre3405* genes were detected in the *cre1*-*96* deletion strain (Fig. 4a–c), whereas *gal1* transcripts accumulated to similar levels (Fig. 4d). Altogether, this suggests that Cre1-96 acts directly on *xyr1* transcript formation and thereby indirectly influences the transcript levels of *xyl1*, *bga1* and *Tre3405*.

### Cre1-96 fulfils the requirements for a transcription factor

**Fig. 5:**
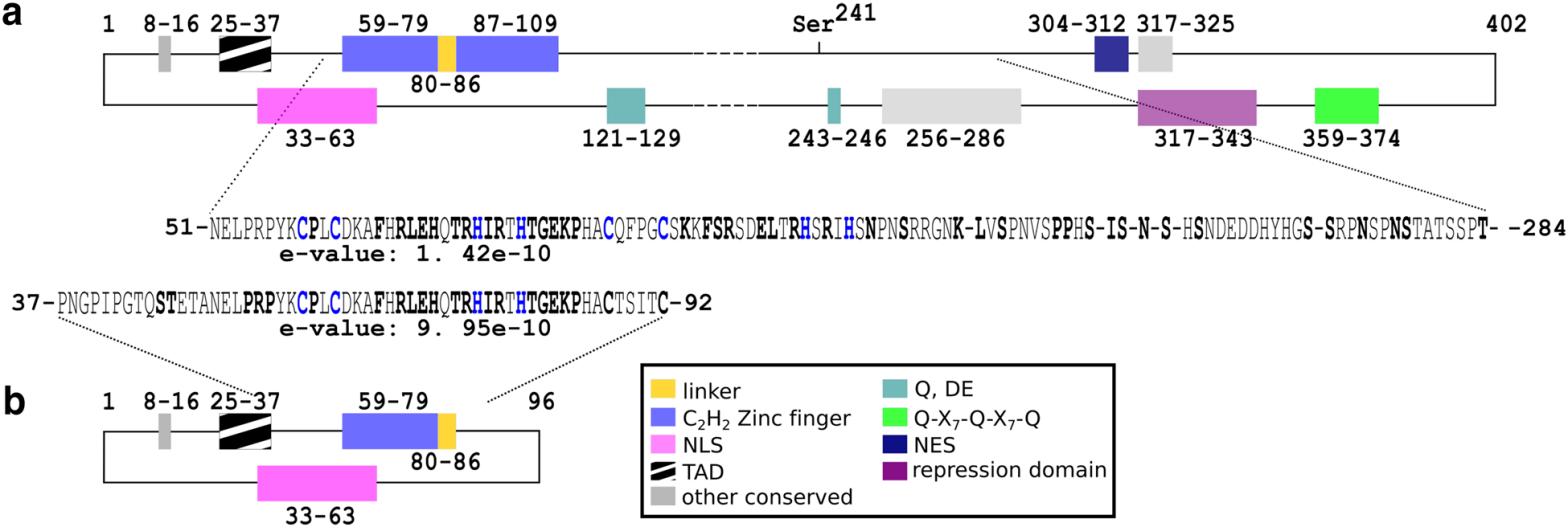
*In silico* domain prediction of Cre1 (**a**) and Cre1-96 (**b**). The putative domains of Cre1 (A) and Cre1-96 (B) were predicted by a number of in silico prediction tools and alignment algorithms as described in the Methods section. Numbers indicate the amino acid (aa) positions and coloured boxes indicate identified domains: blue, C_2_H_2_ zinc finger; yellow, linker; pink, nuclear localization signal (NLS); black-white striped, transactivation domain (TAD); grey, other conserved region; turquoise, Q (glutamine), DE (aspartic (D) and glutamic acid (E)); green, Q-X_7_ Q-X_7_-Q; dark blue, nuclear export signal (NES) and violet, repression domain. Phosphorylation site Ser241 according to [20] is marked. The predicted zinc finger domain superfamily are presented as amino acid sequence and the zinc finger binding sites (cysteine (C) or histidine (H)) are coloured in blue. The e-value is given below the amino acid sequence

Enzymatic measurements and transcript analysis suggested that Cre1-96 exerts a positive effect on cellulase and xylanase gene expression, on the transcript formation of cellulase and xylanase-encoding genes, and most importantly on *xyr1*. To be considered as an activator, some properties need to be fulfilled. First of all, Cre1-96 needs to bind the DNA of its target genes, which is supported by previously reported in vivo footprinting experiments and in vitro protein-DNA binding studies for Cre1-96 and Cre1 [7, 17, 20]. A second essential prerequisite is its localization in the nucleus, at least transiently. In silico domain analysis revealed that Cre1-96 has a putative bipartite nuclear localization signal (NLS) (TVIK – linker – RPYK) located at amino acids (aa) positions 33–63 (Fig. 5a). This bipartite NLS was found with a score of 5 in the case of both proteins, Cre1 and Cre1-96. Scores ranging from 3 to 5 suggest that the protein can be localized both in the nucleus and the cytoplasm. Besides this, alignment of Cre1-96 and Cre1 to homologues from other filamentous fungi revealed further conserved domains or amino acids (Additional file 4: Figure S4). In Cre1-96 a part of a zinc finger domain was identified at 59–79 aa, and a putative transactivation domain (25–37 aa) as well (Fig. 5b). The conserved sequence parts, which are missing in Cre1-96 compared to the full-length Cre1, are a part of the full zinc finger binding domain (87–109 aa), stretches of acidic amino acids (121–129 aa, 243–246 aa, 359–374 aa), two other conserved domains (256–289 aa and 317–325 aa), the nuclear export signal (NES, 304–312 aa), a C-terminal repression domain (317–343 aa) and the phosphorylation site at Ser241 [20] (Fig. 5a). To summarize the in silico analysis, the truncated protein Cre1-96 has lost many potentially important domains but still contains all domains that are essential for a transcription factor, i.e. a DNA-binding domain, NLS and one N-terminal acidic region potentially functioning as activator domain.

**Fig. 6:**
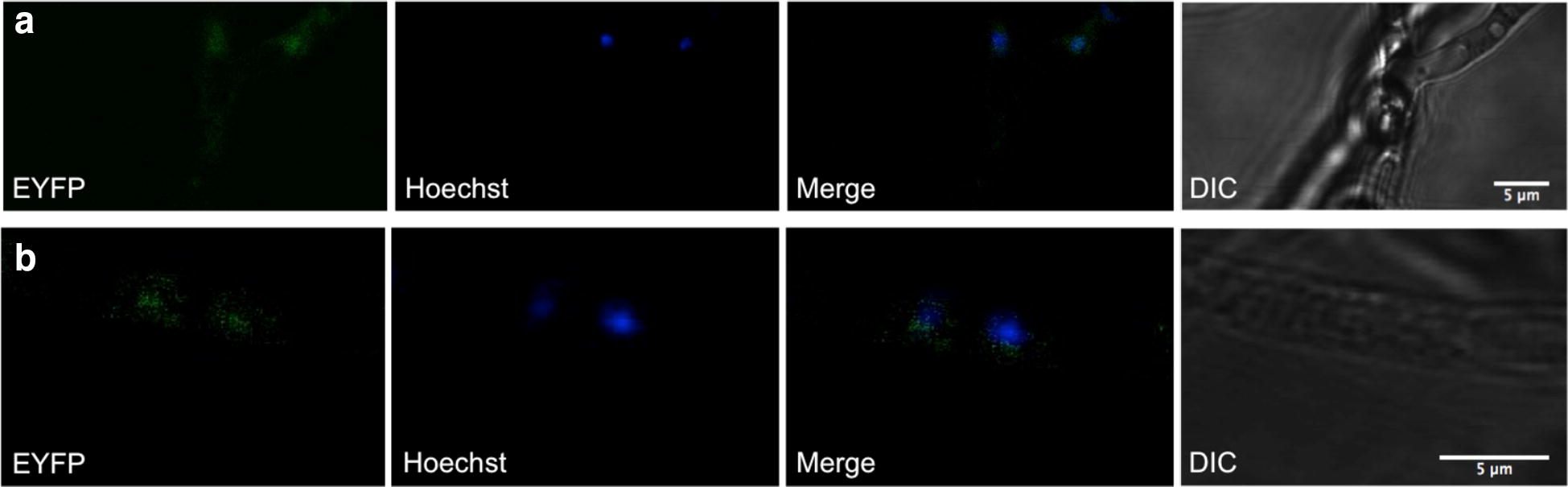
Confocal microscopy analysis of the localization of Cre1-96. The *T. reesei* strain QM6a*cre1*-*96::eyfp* was cultivated in liquid medium supplemented with 1% (w/v) d-glucose (**a**) or 1% (w/v) lactose (**b**). For the visualization of fungal nuclei a Hoechst staining was performed. The following pictures were imaged to localize Cre1-96::eYFP in the fungal hyphae: detection of eYFP (EYFP), detection of the nuclei within the fungal cells (Hoechst), overlay of eYFP and Hoechst emissions (Merge), and brightfield image (DIC). Scales are given in the DIC pictures

To monitor the localization of Cre1-96 in the fungal hyphae, a strain expressing an eYFP-tagged Cre1-96 was generated and cultivated in liquid medium containing d-glucose or lactose. It should be noted that we used QM6a for analysis of the nuclear transport of Cre1-96 to exclude any cross-genetic effects resulting from the other mutations present in Rut-C30. Confocal fluorescence microscopy was performed using a droplet of the liquid culture and the visualization of the fungal nuclei was achieved with Hoechst staining. The localization of Cre1-96 was determined by the detection of fluorescence emission of eYFP. Merging of the eYFP signal and the nuclei fluorescence emissions revealed the presence of Cre1-96 in the nuclei of *T. reesei*. Nuclear localization of Cre1-96 was observed under both, repressing (Fig. 6a) and inducing conditions (Fig. 6b), similar to the full length Cre1 [21].

### Cre1-96 targets Cre1-binding sites within the URR of *xyr1*

**Fig. 7:**
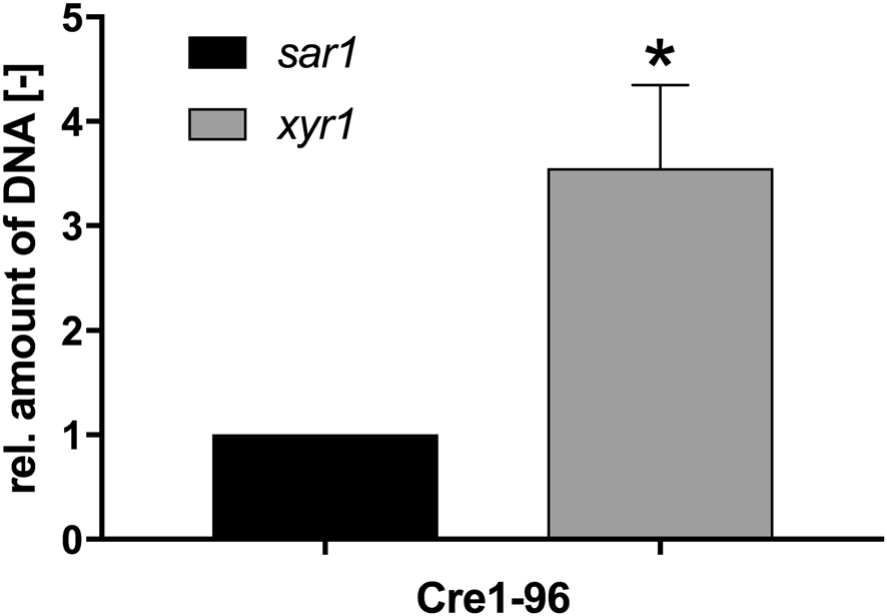
Chromatin immunoprecipitation of Cre1-96::eYFP. The *T. reesei* strain QM6a*cre1*-*96::eyfp* was cultivated for 16 h in 20 mL MEX medium supplemented with 1% (w/v) d-glucose. After crosslinking, the chromatin was enzymatically fragmented by MNase treatment and Cre1-96::eYFP targeted DNA was enriched using anti-GFP antibodies. The relative amount of DNA was measured by qPCR. The immunoprecipitated DNA was normalized to the input control. The resulting ratio for the *xyr1* gene (grey bar) was further normalized to the ratio of housekeeping gene *sar1*, which is set to 1 (black bar). The values were statistically analysed in a confidence interval of 95% and the asterisk indicates a significant difference

To learn where the transcription factor Cre1-96 is targeted to, we performed chromatin immunoprecipitation (ChIP) followed by qPCR analyses. For this purpose, the strain expressing eYFP-tagged Cre1-96 was used. As an initial control we tested cellulase activities and biomass formation in tagged and untagged strains to exclude any impact of the eYFP-tag. The results of these preliminary experiments show no impact of the tag on Cre1-96 function (Additional file 5: Figure S5). As the nuclear localization of Cre1-96 was observed after 16 h cultivation in liquid malt extract (MEX) medium supplemented with d-glucose, this condition was chosen for the ChIP experiment. An enrichment of Cre1-96 was identified with anti-GFP antibodies (please note that they are able to bind eYFP) and qPCR. Since we had already indications that *xyr1* is a target of Cre1-96, specific primers were chosen for the analysis of Cre1-96 associated DNA within the URR of *xyr1*. The relative amount of Cre1-96 targeted DNA is almost threefold enriched in this target region compared to the non-target housekeeping gene *sar1* indicating that indeed the truncated Cre1-96 protein might directly activate *xyr1* transcription (Fig. 7).

### Chromatin accessibility is only moderately affected by a *cre1*-*96* deletion

**Fig. 8:**
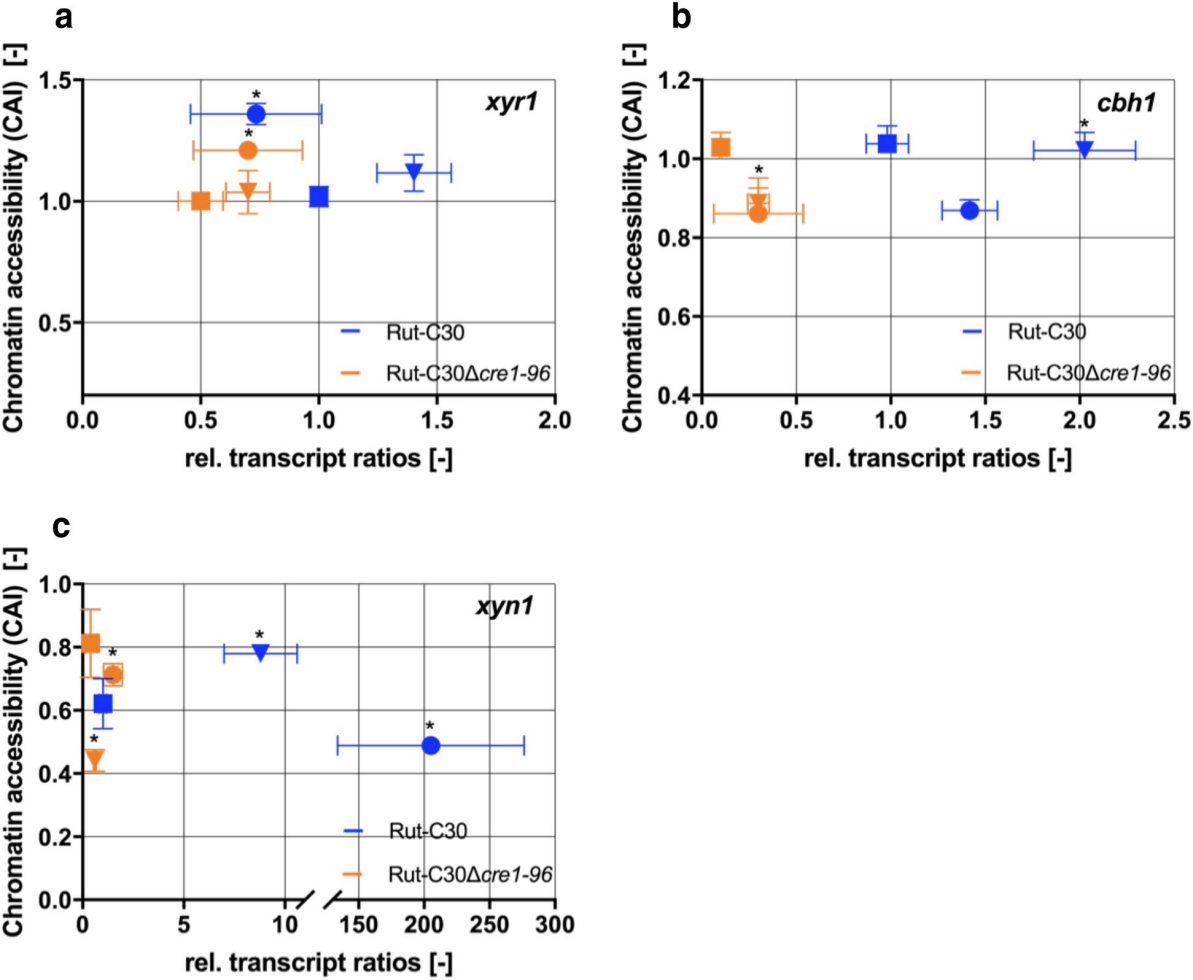
Chromatin accessibility under cellulase inducing conditions*. T. reesei* strains Rut-C30 (blue symbols) and Rut-C30Δ*cre1*-*96* (orange symbols) were cultivated in liquid medium supplemented with 1% (w/v) lactose for 36 (squares), 48 (triangles) and 60 h (dots). CHART-PCR and transcript analysis of *cbh1* (**b**), *xyn1* (**c**) and *xyr1* (**a**) were performed in biological and technical replicates. Both sets of data were normalized to the housekeeping genes *sar1* and *act*. The relative transcript ratios are given as means and plotted on the x-axis and the chromatin accessibility indices (CAI) are plotted on the y-axis. Error bars indicate the standard deviations. All CAI values were statistically analysed in a confidence interval of 95% and asterisks indicate significant differences amongst the chromatin-related data

Previous reports demonstrated a role of Cre1-96 in promoting chromatin accessibility [17]. Hence, we analysed the chromatin accessibility in the URR of Cre1-96 target genes (i.e. *xyr1*, *xyn1* and *cbh1)* in the *cre1*-*96* deletion strain and its parent strain under inducing conditions. Both strains were cultivated in liquid medium on lactose. The fungal mycelium was harvested after 36, 48 and 60 h, followed by chromatin accessibility real-time PCR (CHART-PCR). In the case of *xyr1*, significant differences in the chromatin accessibility were just found after 60 h (Fig. 8a). However, there is no relation between the chromatin status and the transcript level suggesting that chromatin accessibility as measured by our assays is not changing with transcriptional activity. In the case of *cbh1*, significant opening of chromatin in Rut-C30 went along with higher transcript level compared to the deletion strain (Fig. 8b). However, this could be only observed for one time point (i.e. 48 h). Finally, the chromatin accessibility in the *xyn1* URR did differ between Rut-C30 and the *cre1*-*96* deleted strain at two time points of investigation (Fig. 8c). However, again a transcription-related change in accessibility could not be observed.

### Constitutively expressed *cre1*-*96* enhances cellulase activity

**Fig. 9:**
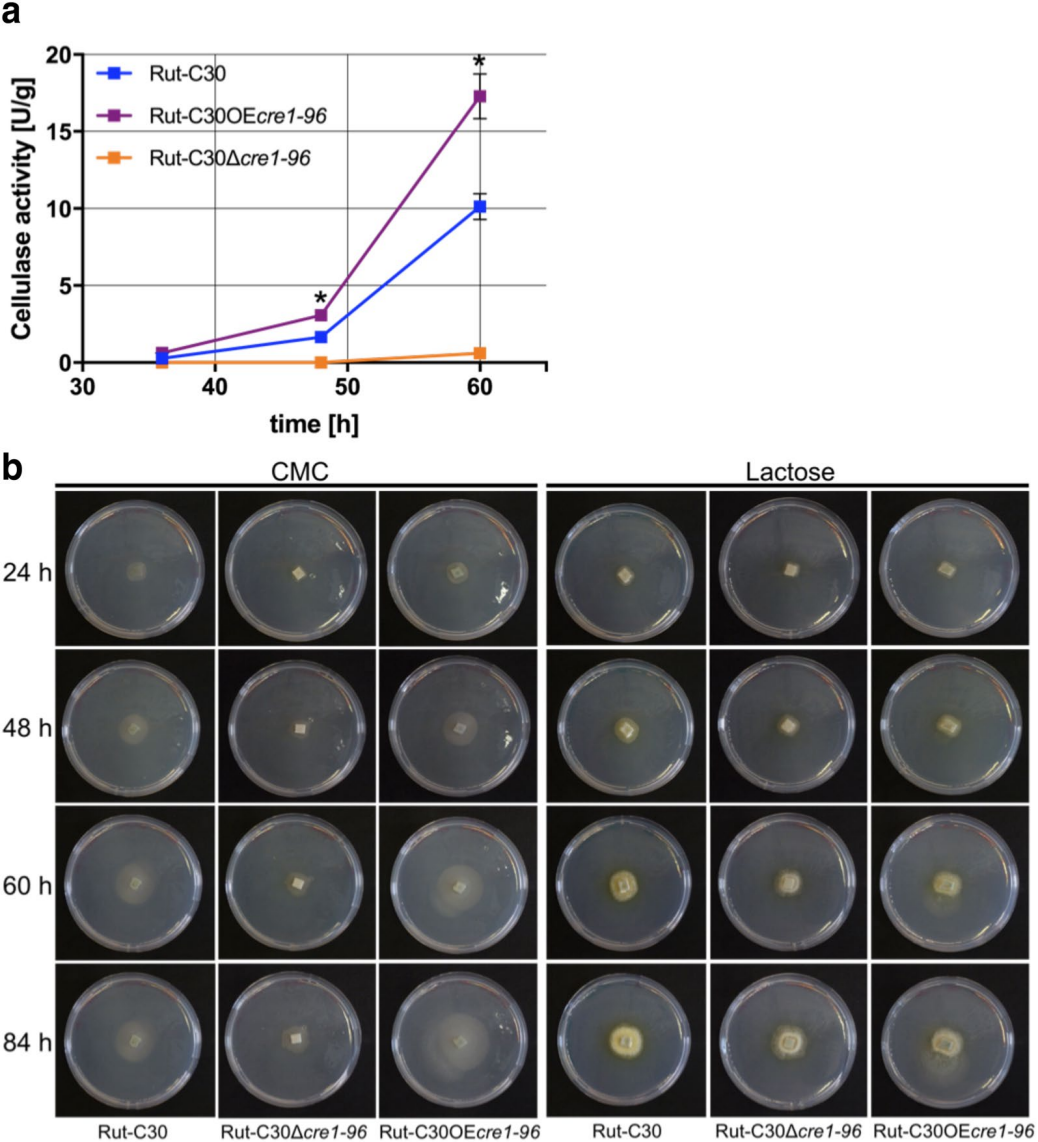
Cellulase activity in presence of a constitutively expressed Cre1-96. **a** *T. reesei* strains Rut-C30 (blue squares), Rut-C30OE*cre1*-*96* (purple squares) and Rut-C30Δ*cre1*-*96* (orange squares) were cultivated in liquid medium supplemented with 1% (w/v) lactose for 36, 48 and 60 h. The endo-cellulolytic activities in the culture supernatants were measured in biological and technical duplicates and normalized to the biomass. The enzymatic activities are given as means and the error bars indicate the standard deviations. Error bars are not shown for standard deviations ≤ 3.5%. The values were statistically analysed by an ordinary one-way ANOVA and a Tukey’s posthoc test in a confidence interval of 95%, and asterisks indicate significant differences. **b** For the growth assays, the *T. reesei* strains Rut-C30, Rut-C30OE*cre1*-*96* and Rut-C30Δ*cre1*-*96* were pre-grown on MEX plates and were then transferred to MA medium plates supplemented with 1% (w/v) CMC or 1% (w/v) lactose. Plates were incubated at 30 °C and pictures were taken after 24, 48, 60 and 84 h

Based on above findings, we have solid indication that Cre1-96 is a necessary activating regulator for cellulase gene expression in Rut-C30. For benefits towards biotechnological applications, we constructed a *T. reesei* strain having a constitutively expressed *cre1*-*96* under the control of the *tef1* promoter (in the following termed Rut-C30OE*cre1*-*96)*, which was cultivated in parallel with the deletion strain and the parent strain in liquid medium on lactose. Cellulase activities were subsequently measured in the culture supernatants. Rut-C30OE*cre1*-*96* had a constant increase in the cellulase activity over time, and it was significantly higher than in the other two strains from 48 h of incubation time on (Fig. 9a). After 60 h of incubation, Rut-C30OE*cre1*-*96* outcompeted Rut-C30 in cellulolytic performance almost twofold. With regard to the growth on cellulase inducing substrates, we observed a similar (24 and 48 h) or a slightly faster growth (60 and 84 h) of Rut-C30OE*cre1*-*96* compared to Rut-C30 on CMC plates (Fig. 9b). On lactose, no visible differences in colony size were observed amongst all three strains (Fig. 9b). Similar growth was observed under non-inducing (glycerol) and repressing conditions (d-glucose) (Additional file 6: Figure S6). Importantly, Rut-C30OE*cre1*-*96* did not show the non-radial growth that was observed for Rut-C30Δ*cre1*-*96* (Additional files 7 and 8: Tables S1 and S2).

## Discussion

The carbon catabolite repressor Cre1 represses transcription of its targets genes by binding their URR. Surprisingly, if truncated, Cre1-96 is still able to bind DNA but converts to a putative activator. Like Cre1/Cre1-96, the transcription factor PacC, which is involved in pH-regulation in *A. nidulans* [22], is a zinc finger protein. PacC is processed by the Pal signalling pathway under alkaline pH and subsequently, moves into the nucleus. In its truncated form it acts as repressor of acidic-expressed genes. Even if its molecular action, namely competition for DNA binding, is different from Cre1-96, in both cases a truncated zinc finger protein acts as transcription factor. The detailed findings of the Cre1 repressor truncation in *T. reesei* are discussed below.

When *cre1* is exchanged for *cre1*-*96* in the wild-type strain QM6a, higher *cbh1*, *cbh2* and *xyr1* transcript levels were obtained compared to a full deletion of *cre1* [17]. However, Rut-C30 that natively carries Cre1-96 had even higher transcript levels of those genes than the QM6a-CREI_96_ strain. Therefore, we deleted *cre1*-*96* in Rut-C30 to study the effects on transcript and corresponding enzyme levels.

We observed growth deficiencies, i.e. a slower growth, reduced biomass formation and growth abnormalities, in the Rut-C30 strain lacking *cre1*-*96*. Under cellulase inducing conditions, we found reduced growth in the *cre1*-*96* deletion strain compared to its parental strain on CMC plates and lactose liquid cultures (compare Figs. 1, 2a) and significant differences in the transcript ratios of genes involved in the lactose metabolism (compare Fig. 4). This indicates a change either in uptake of degradation products into the cell by transporters or in the enzymatic activity required for the conversion of CMC or lactose into an inducing substance (e.g. transglycosylation by BGLI). We did not observe differences in growth on lactose between the parent and the *cre1*-*96* deletion strain in the case of cultivation on plates while we did observe differences in the case of cultivation in liquid medium (i.e. determination of the mycelial biomass weight). Interestingly, Cánovas and colleagues found that the biomass accumulation from plates does usually not correlate with the radial growth diameter [23] so these abnormalities cannot be explained by the current model. Particularly, a non-radial growth of fungal hyphae was observed on lactose in the *cre1*-*96* deletion strain (compare Fig. 1). At this point it has to be mentioned that, a *cre1* deletion in the *T. reesei* strain QM6a leads to strongly impaired growth and morphological changes [8]. Portnoy and colleagues identified several genes involved in hyphal development (e.g. RAS1, PhiA, MedA), which are regulated by Cre1 on d-glucose [10]. Also in other filamentous fungi, like *Neurospora crassa* (*N. crassa*), CRE-1 seems to influence the hyphal growth and polarity because the enzyme activity of an involved cAMP protein kinase A is dependent on CRE-1 [24]. Altogether, this implies that Cre1-96 might exert additional functions (similar to Cre1), besides its role in cellulase and hemicellulase gene expression. Another aspect worth considering was reported by dos Santos Castro and colleagues. RNA sequencing analysis of *T. reesei* QM9414 under repressing (d-glucose) and inducing (cellulose, α-sophorose) conditions [25] indicated that several MFS permeases are differentially expressed on d-glucose. Notably, amongst the strongest down-regulated genes in the absence of Cre1 are proteins involved in cellular transport, such as MFS permeases [10]. This indicates that Cre1 and most probably Cre1-96 might also play a role in the sugar uptake in the cell.

With regard to the cellulase and xylanase activity, we observed either a loss or strong reduction in enzymatic activities in the *cre1*-*96* deletion strain compared to its parental strain (compare Fig. 2b, c). In Rut-C30, the transcript profile of *xyr1* relates to the profile of *cbh1* (compare Fig. 3), which is in full agreement with previously published results [26]. Most interestingly, the transcription profile of *cre1*-*96* relates to the profile of *xyr1* (compare Figs. 2d, 3c). Thus, Cre1-96 might have an effect on the regulation of *xyr1* transcription.

The fluorescence microscopy revealed that Cre1-96 is under repressing and inducing conditions present in the nucleus. A carbon source-dependent shuttling of Cre1 between cytosol and nucleus was proposed by Lichius and colleagues [21]. In silico analysis suggests that Cre1 has a nuclear export signal (NES) at amino acids positions 304–312 (LPSLRNLSL, predicted by using [27]). Cre1-96 lacks this putative NES due to its truncation and therefore, could remain inside the nucleus regardless the used carbon source. Besides this, Cre1-96 possesses a putative N-terminal transactivation domain as Cre1 does, but Cre1-96 importantly lacks the C-terminus of Cre1 (compare Fig. 5) that highly likely mediates repression as it was described for CreA in *Aspergillus nidulans* [28]. Taken together the extended residence in the nucleus, the presence of a putative transactivating domain and the lack of the repression domain, would explain the positive impact of Cre1-96 on the cellulase activity in Rut-C30 compared to the *cre1*-*96* deletion strain under cellulase inducing conditions.

Notably, Cre1-96 also lacks the previously identified phosphorylation site Ser241 [20]. In the case of Cre1, Ser241 needs to be phosphorylated for an efficient DNA binding under repressing conditions. However, Czifersky and colleagues reported that GST fusion proteins of Cre1 fragments without Ser241 bind in vitro regardless the tested condition. Besides this, previously published in vivo footprinting results also supported the capability of Cre1-96 to bind DNA [17].

With regard to the targeting of Cre1-96, we found that Cre1-96 is enriched on its DNA binding sites in the *xyr1* URR. However, the chromatin accessibility in the *xyr1* URR is not significantly different. Neither for *cbh1* or *xyn1* any cohesive trend could be observed. Noteworthy, an earlier reported nucleosomal mapping of *cbh1* and *cbh2* promoter regions showed no positioned nucleosomes under repressing and inducing conditions in Rut-C30 [29, 30]. This lack of positioned nucleosomes is the likely explanation of the similarity in DNA accessibility observed in our experiments between conditions of expressed and non-expressed genes.

Anyhow, we propose here that a truncation of Cre1 positively influences the transactivator Xyr1 and thus enhances cellulolytic performance and phenotypically converts the carbon catabolite repressor into an activator.

## Conclusions

Due to a truncation the Cre1 repressor can turn into an activator as seen in Cre1-96, which now functions to activate cellulase and xylanase expression. Cre1-96 meets all requirements for a transcription factor. It localizes to the nucleus and directly binds to the URR of target genes, in particular of the main transactivator of the mentioned enzymes, Xyr1, and most probably exerts thereby its activation role. Our findings encourage testing this strategy to increase enzymatic performance in other filamentous fungi, which contain functional Cre1 homologues.

## Methods

### Fungal strains

The *T. reesei* strains QM6aΔ*tmus53* [31], the QM6aΔ*tmus53* bearing an eYFP-tagged Cre1-96 (referred to in the text as QM6a*cre1*-*96::eyfp*, this study), Rut-C30Δ*tmus53* (referred to in the text as Rut-C30, VTT Finland), two *cre1*-*96* deletion strains Rut-C30Δ*tmus53*Δ*cre1*-*96* (referred to in the text as Rut-C30Δ*cre1*-*96* (1) and (2), this study) and the *cre1*-*96* constitutively expressing strain Rut-C30Δ*tmus53*OE*cre1*-*96* (referred to in the text as Rut-C30OE*cre1*-*96*, this study) were maintained on malt extract (MEX) agar plates containing 0.1% (w/v) peptone from casein at 30 °C. Uridine was added to a final concentration of 5 mM for all Rut-C30 strains. For strain selection hygromycin B was added to a final concentration of 113 U/mL for the QM6a-related strains and 56.5 U/mL for the Rut-C30-related strains. Homokaryon selection was carried out on MEX/peptone/hygromycin B plates, with uridine if applicable, and 0.1% (w/v) Igepal C-60.

### Growth conditions

If not indicated otherwise in a Methods section, for cultivation experiments 10^6^ conidia spores per mL were incubated in 100 mL Erlenmeyer flasks on a rotary shaker (180 rpm) at 30 °C for 60 h in 30 mL of MA medium supplemented with 0.1% (w/v) peptone and 1% (w/v) lactose or 1% (w/v) d-glucose as sole carbon source. If not stated otherwise, all strains were cultivated in triplicates and were harvested after 36, 48 and 60 h of cultivation. Fungal mycelia were separated from the supernatant by filtering with Miracloth (EMD Millipore, part of Merck KGaA, Darmstadt, Germany). Mycelia grown on lactose were weighted immediately before shock freezing and wet weight was used as the biomass reference for the enzymatic assays. Frozen mycelia were used for genomic DNA extraction, RNA extraction and for chromatin digestion. Culture supernatants were used for the measurement of enzymatic activities in technical duplicates.

For the comparison of the growth behaviour on plates, the strains Rut-C30, Rut-C30Δ*cre1*-*96* (1) and (2) and Rut-C30OE*cre1*-*96* were pre-grown on solid MEX media with 0.1% (w/v) peptone and were transferred in biological duplicates onto MA agar plates supplemented with 1% (w/v) lactose, CMC, xylan, glycerol or d-glucose for 84 h. Due to the same growth behaviour of the duplicates, only one replicate is displayed in the figures. In the case of the deletion strains, Rut-C30Δ*cre1*-*96* (2) is shown.

### Plasmid construction

*Escherichia coli* strain Top10 (Invitrogen, part of Life Technologies, Paisley, UK) was used for all cloning purposes throughout this study and grown on LB medium at 37 °C. Generation of competent *E. coli* cells and subsequent transformation was performed according to standard protocols using CaCl_2_. If applicable, ampicillin and hygromycin B were added to final concentrations of 100 mg/mL and 113 U/mL, respectively.

PCRs for all cloning purposes were performed with Pwo DNA Polymerase (peqlab VWR, Radnor, Pennsylvania, USA) or Phusion High-Fidelity DNA Polymerase (Thermo Scientific, Waltham, Massachusetts, USA) according to the manufacturer’s instructions. All used primers were purchased from Sigma Aldrich and are listed in Table 1.

**Table 1:** Primers used for strain construction in this study

Primer name	Sequence 5′–3′	References
5′cre1_NotI fwd	GCGGCCGCTGGAGGTGACGAGAAGAAAAATTCAGG	This study
5′cre1_XmaI rev	CCCGGGAGTCAAAAAGCAAGTACGCGACGTTG	This study
hph_XmaI_BamHI	CCCGGGTTGGATCCAGGGAGACGAGGTTGTGATGAATAC	This study
hph_SpeI rev	ACTAGTAAGTAGCACCGCTGTCGTCTG	This study
5Pcre1_NotI fwd	GCGGCCGCAGCCAAGACTCAGCATAAAGAGGTTG	This study
5Pcre1_XmaI rev	CCCGGGAGGTACCAAACAAAGCGAGCAAGTAC	This study
ptef_BspEI fwd	TCCGGATGTGTGACAGCTCGCGCAG	This study
ptef_NdeI rev	CATATGTGACGGTTTGTGTGATGTAGCGTG	This study
cre1-96_NdeI fwd	CATATGATGCAACGAGCACAGTCTGCC	This study
Cre1-96_BamHI rev	GGATCCTTAGAAAAAAAAGCAGGTAATGGAGGTGC	This study
cre1-96_BspEI fwd	TCCGGAATGCAACGAGCACAGTCTGCC	This study
cre1-96-TAA_NdeI rev	CATATGGAAAAAAAAGCAGGTAATGGAGGTGC	This study
linker_NheI rev	GCTAGCGCGGGGGGCGCAC	This study
linker_NdeI fwd	CATATGCACAACATGGTCAAGCAGAAGC	This study
YFP_NheI fwd	GCTAGCATGGTCAGCAAGGGCGAGG	This study
YFP_BamHI rev	GGATCCCTTGTACAGCTCGTCCATGCCG	This study

For the construction of the *cre1*-*96* deletion cassette the 5′-flank of *cre1*-*96* was amplified by PCR using chromosomal DNA of *T. reesei* QM6aΔ*tmus53* (identical sequence to Rut-C30) as template with the primers 5′cre1_NotI fwd and 5′cre1_XmaI rev. The PCR product was subcloned into pJET1.2 (Thermo Scientific) by blunt end ligation using T4 DNA ligase (Thermo Scientific) yielding pJET1.2-5′-cre1. The 3′-flank of *cre1* and a hygromycin B resistance cassette were amplified by PCR using chromosomal DNA of *T. reesei* QM6a-Cre1_96_ [17] as template with the primers hph_XmaI_BamHI and hph_SpeI rev and was inserted into pJET1.2 by blunt end ligation yielding pJET1.2-hph. The hygromycin B resistance cassette bears the constitutive promoter of the *pki* gene, the hygromycin B structural gene and the terminator of *cbh2* [32]. The subcloned 5′-flank of *cre1* was recovered by NotI/XmaI digestion and was inserted into the NotI/XmaI-digested vector pJET1.2-hph. The resulting plasmid was termed pJET1.2-5hph3cre1. Subsequently, the vector pJET1.2-5hph3cre1 was cut by NotI and SpeI and the cassette was inserted into a NotI/SpeI-digested derivative pMS plasmid yielding pMS*-5hph3cre1. The orientation of the hygromycin B resistance gene and the *pki* promoter were in the opposite orientation as the 5′-flank and the 3′-flank of *cre1*. This was determined by plasmid sequencing (Microsynth, Balgach, Switzerland).

For the constitutive expression of *cre1*-*96* the promoter of the *tef1* gene was used. For this purpose, the promoter region (1500 bp upstream of ATG) of *tef1* (p*tef1*) was amplified by PCR using chromosomal DNA of *T. reesei* QM6aΔ*tmus53* as template with the primers ptef_BspEI fwd and ptef_NdeI rev. The structural gene *cre1*-*96* was amplified using the primers cre1-96_NdeI fwd and cre1-96_BamHI rev. Both PCR fragments were subcloned into pJET1.2 (Thermo Scientific), yielding pJET1.2-Ptef and pJET1-2-cre1-96. Both plasmids were digested by BspEI/NdeI, the p*tef1* fragment was isolated and ligated into the BspEI/NdeI-digested pJET1.2-cre1-96 to yield pJET1.2-Ptefcre1-96. The 5′-flank of *cre1* started at -1500 bp until 2400 bp to avoid a residual background of the native *cre1* promoter. This 5′-flanking region was amplified using the primers 5Pcre1_NotI fwd and 5Pcre_XmaI rev and was subcloned into pJET1.2 by blunt end ligation using T4 DNA ligase (Thermo Scientific) yielding pJET1.2-5Pcre1. The 3′-flank of *cre1* and a hygromycin B resistance cassette was constructed as described for the *cre1*-*96* deletion cassette. The subcloned 5′-flank of *cre1* was recovered by NotI/XmaI digestion of pJET1.2-5Pcre1 and was inserted into the NotI/XmaI-digested vector pJET1.2-hph. The resulting plasmid was termed pJET1.2-5′cre1-hph. The plasmid pJET1.2-Ptefcre1-96 was BspEI/BamHI-digested, the fragment Ptefcre1-96 isolated and ligated by cohesive ends with a XmaI/BamHI-digested pJET1.2-5′cre1-hph to yield pJET1.2-3Ptefcre1-96. Subsequently, this plasmid was cut by NotI and SpeI and the cassette was inserted into a NotI/SpeI-digested derivative pMS plasmid yielding the final plasmid pMS*-Ptefcre1-96. The correct orientation and sequence of the plasmid were confirmed by sequencing (Microsynth).

For construction of pMS*-*cre1*-*96::eyfp* the coding sequence of *cre1*-*96*, a linker and *eyfp* were amplified by PCR using chromosomal DNA from *T. reesei* QM6a-Cre1_96_ and the plasmid pCD-EYFP [33] as templates and the following primers: cre1-96_BspEI fwd and cre1-96-TAA_NdeI rev to amplify *cre1*-*96* from QM6a-Cre1_96_; linker_NdeI fwd and linker_NheI rev to amplify the linker sequence from the pCD-EYFP plasmid; YFP_NheI fwd and YFP_BamHI to amplify the coding sequence of *eyfp* from the pCD-EYFP plasmid. Importantly, the fluorescent tag was fused to the C-terminus of the Cre1-96 because this was reported to be necessary for proper recruitment and import in the case of the full length Cre1 [21]. The PCR products were subcloned into pJET1.2, yielding pJET1.2-cre1-96(-TAA), pJET1.2-linker and pJET1.2-YFP. The first two plasmids were digested with BspEI/NdeI, the cre1-96 fragment was isolated and ligated into the BspEI/NdeI-digested recipient vector pJET1.2-linker to generate the plasmid pJET1.2-cre1-96-linker. The insert cre1-96-linker was recovered by BspEI/NheI digestion and cloned into the BspEI/NheI-digested pJET1.2-YFP to yield pJET1.2-cre1-96::eyfp. A BspEI/BamHI double digest of pJET1.2-cre1-96::eyfp recovered the cre1-96::eyfp insert, which was cloned into the XmaI/BamH-digested vector pJET1.2-5hph3cre1 yielding pJET1.2-5cre1-96::eyfp. Finally, the 5cre1-96::eyfp was recovered by NotI/SpeI digestion of pJET1.2-5cre1-96::eyfp and was cloned into a NotI/SpeI-digested derivative pMS plasmid yielding the final plasmid.

### Fungal protoplast transformation

Protoplast transformation of *T. reesei* was performed as described by Gruber et al. [34]. For the gene replacement of *cre1,* the plasmid pMS*-*cre1*-*96::eyfp* was linearized by NotI digestion and transformed into *T. reesei* QM6aΔ*tmus53*. For the deletion of *cre1*-*96*, the plasmid pMS*-5hph3cre1 was linearized by NotI digestion and transformed into *T. reesei* Rut-C30Δ*tmus53*. For the constitutive expression of *cre1*-*96* under the control of the promoter of *tef1,* the plasmid pMS*-*ptef*::*cre1*-*96* was transformed into *T. reesei* Rut-C30Δ*tmus53*. Each transformation reaction was added to 40 mL melted, 50 °C warm MEX agar containing 1.2 M D-sorbitol. This mixture was poured into 4 sterile petri dishes, which were incubated at 30 °C for at least 2 h for protoplast regeneration. Appropriate amount of hygromycin B was added to 40 mL melted, 50 °C warm MEX agar containing 1.2 M D-sorbitol and was poured as a 10 mL-overlay on all 4 plates. Transformation plates were further incubated at 30 °C for 2–4 days until colonies were visible. The resulting candidates were subjected to 3 rounds of homokaryon selection by streaking.

### Isolation of genomic DNA

Genomic DNA was isolated from approximately 50 mg mycelium in 1 mL CTAB buffer (1.4 M NaCl, 100 mM Tris–HCl pH 8.0, 10 mM EDTA, 2% (w/v) CTAB) by homogenization using a FastPrep(R)-24 cell disrupter (MP Biomedicals, Santa Ana, California, USA) followed by a phenol/chloroform extraction. RNA was degraded using RNaseA (Thermo Scientific). DNA was precipitated with isopropanol, washed with 70% (w/v) ethanol, and dissolved in distilled H_2_O.

### Diagnostic PCR analysis

100 ng of chromosomal DNA was used as template in a 25-µL-PCR using GoTaq^®^ G2 polymerase (Promega, Madison, Wisconsin, USA) according to manufacturer’s instructions. Primer sequences are provided in Additional file 7: Table S1 and Additional file 8: Table S2. For subsequent agarose gel electrophoresis of DNA fragments a GeneRuler 1 kb DNA Ladder (Thermo Scientific) was applied for estimation of fragment size. DNA sequencing was performed at Microsynth.

### RNA extraction and reverse transcription

Fungal mycelia were homogenized in 1 mL of peqGOLDTriFast DNA/RNA/protein purification system reagent (peqlab VWR, Radnor, Pennsylvania, USA) using a FastPrep(R)-24 cell disrupter (MP Biomedicals). RNA was isolated according to the manufacturer’s instructions, and the concentration was measured using the NanoDrop 1000 (Thermo Scientific). Reverse transcription of the isolated mRNA was carried out using the RevertAidTM H Minus First Strand cDNA Synthesis Kit (Thermo Scientific) according to the manufacturer’s instructions.

### Transcript analysis

Quantitative PCR (qPCR) was performed in a Rotor-Gene Q system (Qiagen, Hilden, Germany). Reactions were performed in technical duplicates or triplicates. The amplification mixture (final volume 15 μL) contained 7.5 μL 2 × iQ SYBR Green Mix (Bio-Rad, Hercules, California, USA), 100 nM forward and reverse primer, and 2.5 μL cDNA (diluted 1:20). Primer sequences and cycling conditions are provided in Table 2. Data normalization using *sar1* and *act* as reference genes and calculations were performed as previously published [35].

**Table 2:** Primer used for qPCR

Primer name	Sequence 5′–3′	References
actfw	TGAGAGCGGTGGTATCCACG	[35]
actrev	GGTACCACCAGACATGACAATGTTG	[35]
sar1fw	TGGATCGTCAACTGGTTCTACGA	[35]
sar1rev	GCATGTGTAGCAACGTGGTCTTT	[35]
xyr1f	CCCATTCGGCGGAGGATCAG	[35]
xyr1r	CGAATTCTATACAATGGGCACATGGG	[35]
taqxyn1 f	CAGCTATTCGCCTTCCAACAC	[13]
taqxyn1 r	CCAAAGTTGATGGGAGCAGAA	[13]
cbh1f	GATGATGACTACGCCAACATGCTG	[12]
cbh1r	ACGGCACCGGGTGTGG	[12]
cre1_a_f	ACCTCCTGAATCCAACGTCGG	[17]
cre1_a_r	TGGGTGCGAATGTGCCTGG	[17]
bga1f	CGTTTGATCCTTTCGGCGGCT	[19]
bga1r	CCAAAGGTCATGTATATGTTGAAGATGGTC	[19]
gal1f	GGAGGCATGGACCAGGC	[19]
gal1r	GACATGCTTGTTGGAGGTGACG	[19]
xorf	CTGTGACTATGGCAACGAAAAGGAG	[19]
xorr	CACAGCTTGGACACGTGAAGAG	[19]
st RT 1	CCGTCTACCGTCTGTTGTGC	[5]
st RT 2	GAAGTAGGAAAGAACCGCATTG	[5]

### In silico prediction of protein domains

The protein sequences of Cre1 (JGI *Trichoderma reesei* QM6a v2.0 Database, Protein ID 120117) and Cre1-96 (JGI *Trichoderma reesei* Rut C-30 v1.0 Database, Protein ID 23706) were obtained from the respective genome databases [36, 37]. The identification of the DNA binding domain (i.e. C_2_H_2_ zinc finger and linker sequence) was achieved by using the NCBI conserved domain search [38]. Multiple sequence alignment of Cre1-96, Cre1 and its homologues of *Aspergillus nidulans* (*A. nidulans*, NCBI Accession ID: XP_663799.1), *Aspergillus niger* (*A. niger*, NCBI Accession ID: XP_001399519.1), *Neurospora crassa* (*N. crassa*, NCBI Accession ID: XP_961994.1), *Trichoderma atroviride* (*T. atroviride*, NCBI Accession ID: XP_013941427.1), *Trichoderma virens* (*T. virens*, NCBI Accession ID: XP_013956509.1) and *Saccharomyces cerevisiae* (*S. cerevisiae*, NCBI Accession ID: NP_011480.1) was conducted using Clustal Omega [39] and identified conserved amino acids and protein domains. Prediction of the NLS was achieved by applying the NLS Mapper [40] on Cre1 and Cre1-96. For the in silico identification of the transactivation domain, the Nine Amino Acids Transactivation Domain (9aaTAD) Prediction Tool [41] was used [42]. As search specification, the less stringent pattern was chosen as the most adequate pattern for both proteins.

### Confocal microscopy

The localization of the eYFP-labelled Cre1-96 was determined by confocal microscopy and image processing using Fiji [43]. Samples were prepared from liquid cultures. Therefore, 10^6^ spores per mL of QM6a*cre1*-*96::eyfp* were used to inoculate 20 mL of MA medium supplemented with 1% (w/v) d-glucose and 1% (w/v) lactose and incubated at 30 °C and 180 rpm for 16 h. A 10-μL sample was taken and embedded between two glass coverslips (24 × 60, 24 × 24). For the nuclear staining, 4 μL of a 1:10-diluted (distilled water) Hoechst 34580 stain (Thermo Scientific, 5 mg/mL in DMSO) was added before putting the glass coverslip on top of the sample and incubated for 10 min in darkness. Live-cell imaging was performed using a Nikon C1 confocal laser scanning unit sitting on top of a Nikon Eclipse TE2000-E inverted microscope base (Nikon Inc., Melville, New York, USA). An argon ion laser emitting a wavelength of 488 nm excited fluorescent proteins and Hoechst stained nuclei. The emission wavelength was detected with a photomultiplier in a range of 500–530 nm. Laser intensity and illumination time were kept the same for all samples. Pictures were taken as a single picture configuration at a resolution of 1024 × 1024 pixels.

### Enzyme assays

Endo-xylanolytic and endo-cellulolytic activities of cultivation supernatants were measured with Xylazyme AX tablet assay and Azo-CMC-Cellulose assay (both Megazyme International Ireland, Wicklow, Ireland) according to the manufacturer’s instructions. For the comparison of cellulolytic activities of Rut-C30OE*cre1*-*96* and the *cre1*-*96* deletion, only Rut-C30Δ*cre1*-*96* (2) was used due to similar results of previous experiments of this study (e.g. transcript analysis).

### Chromatin immunoprecipitation (ChIP) and quantitative PCR analysis

*T. reesei* strain QM6a*cre1*-*96::eyfp* was grown for 16 h in MEX supplemented with 1% (w/v) d-glucose at 30 °C at 180 rpm. Crosslinking was performed with 1% (w/v) formaldehyde for 15 min at room temperature and gentle shaking every 2–3 min. Quenching was performed by the addition of 125 mM glycine at room temperature for 5 min and gently shaking. Mycelia were filtered by Miracloth, washed with distilled water, dry-pressed between sheets of Whatman paper and frozen in liquid nitrogen. The chromatin shearing and the ChIP protocol were performed according to [44] with the following adaptions. An amount of 100–200 mg of fungal mycelia was grinded in liquid nitrogen and suspended in MNase digestion buffer (50 mM Hepes–KOH pH 7.5, 50 mM NaCl, 1 mM CaCl_2_, 5 mM MgCl_2_, 1 mM PMSF, 1× fungal protease inhibitors (Sigma, St. Louis, Missouri, USA)). Chromatin shearing was enzymatically performed by using 0.4 U MNaseI (Sigma,) on 200 μL mycelia aliquots at 37 °C for 13 min. The reaction was stopped by adding 100 μL Lysis Buffer v2 (50 mM Hepes–KOH pH 7.5, 255 mM NaCl, 12 mM EDTA, 2% (w/v) Triton-X100, 0.2% (w/v) Na-deoxcholate, 1 mM PMSF, 1× fungal protease inhibitors (Sigma)). For the precipitation of the protein-antibody complex, an Anti-GFP antibody (ChIP grade; Abcam, Cambridge, UK) and Dynabeads^®^ Protein A magnetic beads (Thermo Scientific) were used. The obtained conjugate was washed 3 times with a low salt buffer (0.1% (w/v) SDS, 1% (w/v) Triton X-100, 2 mM EDTA pH 8.0, 20 mM Tris–HCl pH 8.0, 150 mM NaCl), once with a final wash buffer (0.1% (w/v) SDS, 1% (w/v) Triton X-100, 2 mM EDTA pH 8.0, 20 mM Tris–HCl pH 8.0, 500 mM NaCl) and once with TE buffer. Then, samples were eluted in TES buffer (10 mM Tris–HCl pH 8.0, 1 mM EDTA, 1% (w/v) SDS). Protein-bound DNA was treated with Proteinase K (Thermo Scientific) and DNA samples were purified using the MiniElute PCR Purification Kit (Qiagen) according to the manufacturer’s protocol. The precipitated DNA was quantified by qPCR performed in iCycler Thermal Cycler (Bio-Rad) and the use of a standard curve. A reaction volume of 25 μL including the following compounds: 2× iQ SYBR^®^ Green Supermix (Bio-Rad), 10 μM primers and 5 μL of immunoprecipitated and input DNA (1:5 diluted in EB) or genomic DNA for the standard curve. The annealing temperature was 60 °C and the primer sequences are provided in Table 3. The qPCR cycling protocol and the adequate amounts of reagents were chosen as recommend in the manufacturer’s instructions. All experiments were performed in biological and technical duplicates.

**Table 3:** Primer used for ChIP-qPCR

Primer name	Sequence 5′–3′	Reference
sar1 3UTR f	TGACGGGGAGAACATGTGCTC	This study
sar1 3UTR r	ATGCGACTCCCACAAGTGGTG	This study
ChIP_xyr1 upstream f	TACACAAGAGCAATGGCCCTAGC	This study
ChIP_xyr1 upstream r	TGGATGGATGGAGAACGGGATG	This study

### Chromatin accessibility real-time PCR (CHART-PCR) assays

DNaseI digestions of chromatin and subsequent qPCR analyses were carried out as described before [17]. To be noted, only one of both deletion strains (Rut-C30Δ*cre1*-*96* (2)) was used for this analysis due to similar results from previous experiments of this study (e.g. transcript analysis). The qPCR analyses of the DNaseI-treated samples were performed to measure the relative abundance of DNA of the target regions. PCRs were performed in triplicates in a Rotor-Gene Q system (Qiagen) using the reaction mixture (final volume 20 μL) and the cycling conditions as described before [17]. Primer sequences are provided in Table 4. The amount of intact input DNA of each sample was calculated by comparing the threshold values of the PCR amplification plots with a standard curve generated for each primer set using serial dilutions of genomic, undigested DNA. The chromatin accessibility index (CAI) was defined as: CAI = (Dc1 + Dc2)/2Ds, where Ds is the amount of intact DNA detected for each target region, and Dc1 and Dc2 are the amounts of intact DNA detected for the promoter regions of *sar1* and *act,* respectively, which were used as reference genes for normalization.

**Table 4:** Primer used for CHART-PCR

Primer name	Sequence 5′–3′	References
epiactinTr_f	CTTCCCTCCTTTCCTCCCCCTCCAC	[17]
epiactinTr_r	GCGACAGGTGCACGTACCCTCCATT	[17]
episar1Tr_f	GTCAGGAAATGCCGCACAAGCAAGA	[17]
episar1Tr_r	TGTGTTTTACCGCCTTGGCCTTTGG	[17]
epixyr1_1Tr_f	CCTTTGGCCATCTACACAAGAGCAA	[45]
epixyr1_1Tr_r	CGCAATTTTTATTGCTGTTCGCTTC	[45]
epicbh1_1Tr_f	AAGGGAAACCACCGATAGCAGTGTC	[46]
epicbh1_1Tr_r	TTTCACTTCACCGGAACAAACAAGC	[46]
epixn1_1Tr_f	GCACTCCAAGGCCTTCTCCTGTACT	[46]
epixyn1_1Tr_r	TAGATTGAACGCCACCCGCAATATC	[46]

## Additional files

**Additional file 1: Figure S1.** Deletion of *cre1-96* in *T. reesei* Rut-C30. (A) Rut-C30 was transformed with the plasmid pMS*-5hph3cre1 that bears the deletion cassette consisting of the hygromycin resistance gene under the *pki* promoter and the terminator of *cbh2* (dark grey arrow, *hph*) to replace the native *cre1-96* gene (light grey arrow, *cre1-96*). (B) Agarose gel electrophoresis of diagnostic PCR was performed. Primer pairs added to the respective PCR are indicated on top of the gel, the strain of which the genomic DNA was used as template is indicated below each lane. Candidate strains (Δ*cre1-96* (1) and (2)) yielded expected fragments with the primer pair 1F and 1R or 3F and 3R, and no fragment in case of primer pair 2F and 2R. Rut-C30 was applied as negative control in the case of the PCR using primer pair 1F and 1R and as positive control in the PCR using primer pair 2F and 2R. Water added to the respective PCR in a no template control PCR (NTC). A DNA ladder (L) was included for estimation of fragment size.

**Additional file 2: Figure S2.** Constitutive expression of *cre1-96* in *T. reesei* Rut-C30. (A) Rut-C30 was transformed with the plasmid pMS*-*ptef::cre1-96* that bears the *tef1* promoter (white bar, p*tef1*), the *cre1-96* gene (light grey arrow, *cre1-96*), and the marker cassette (dark grey bar, *hph*). The latter consists of the hygromycin resistance gene under the *pki* promoter and the terminator of *cbh2*. (B) Agarose gel electrophoresis of diagnostic PCR was performed. Primer pairs added to the respective PCR are indicated on top of the gel, the strain of which the genomic DNA was used as template is indicated below each lane. A candidate strain (OE*cre1-96*) yielded expected fragments with all three primer pairs. Rut-C30 was applied as negative control in case of the PCR using primer pair 1F and 1R as well as 2F and 2R and as a positive control in the PCR using primer pair 3F and 3R. A DNA ladder (L) was included for estimation of fragment size.

**Additional file 3: Figure S3.** Growth behaviour of Rut-C30Δ*cre1-96* on glycerol and d-glucose. The *T. reesei* strains Rut-C30 and Rut-C30Δ*cre1-96* (2) were pre-grown on MEX plates and were then transferred to MA medium plates supplemented with 1 % (w/v) glycerol or d-glucose. Plates were incubated at 30 °C and pictures were taken after 24, 48, 60 and 84 hours.

**Additional file 4: Figure S4.** Multiple sequence alignment of Cre1 homologues. Multiple sequence alignment of *T. reesei* Cre1, Cre1-96 and Cre1 homologues of *A. nidulans*, *A. niger*, *N. crassa*, *T. atroviride*, *T. virens* and *S. cerevisiae* was conducted using Clustal Omega (http://www.ebi.ac.uk/Tools/msa/clustalo/). Protein sequences were retrieved from respective genome databases. The alignment revealed conserved amino acids and protein domains based on sequence similarities.

**Additional file 5: Figure S5.** Cellulase activity and biomass formation of QM6*acre1-96* and QM6*acre1-96::eyfp* on d-glucose. The *T. reesei* strains QM6*acre1-96* (blue bar, Cre1-96) and QM6*acre1-96::eyfp* (purple bar, Cre1-96::eYFP) were cultivated in triplicates for 45 hours in MA medium supplemented with 1 % (w/v) d-glucose. Cellulase activities (A) of the culture supernatants were measured in technical duplicates and the biomass (B) was collected by filtration with miracloth and is depicted as dry weight on the y-axis.

**Additional file 6: Figure S6.** Growth behaviour of Rut-C30OE*cre1-96* on glycerol and d-glucose. The *T. reesei* strains Rut-C30, Rut-C30Δ*cre1-96* (2) and Rut-C30OE*cre1-96* were pre-grown on MEX plates and were then transferred to MA medium plates supplemented with 1 % (w/v) glycerol and d-glucose. Plates were incubated at 30 °C and pictures were taken after 24, 48, 60 and 84 hours.

**Additional file 7: Table S1.** Primers used for the diagnostic PCR of Rut-C30Δ*cre1-96* (1) and (2).

**Additional file 8: Table S2.** Primers used for the diagnostic PCR of Rut-C30OE*cre1-96*.

## Abbreviations

aa: amino acid
CAI: chromatin accessibility index
CCR: carbon catabolite repression
CHART-PCR: chromatin accessibility real-time PCR
ChIP: chromatin immunoprecipitation
CMC: carboxymethylcellulose
Cre1: carbon catabolite repressor 1
CreA: carbon catabolite repressor A
EB: elution buffer
eYFP: enhanced yellow fluorescent protein
LB: lysogeny broth
MA: Mandels-Andreotti
MEX: malt extract
NES: nuclear export signal
NLS: nuclear localization signal
qPCR: quantitative PCR
TAD: transactivation domain
URR: upstream regulatory regions
Xyr1: xylanase regulator 1

## References

[1] Singh A, Mishra P. Overview of problems and potential. In: Microbial pentose utilization—current applications in biotechnology. vol. 33; 1995. p. 1–3.

[2] Kumar R, Singh S, Singh OV (2008). Bioconversion of lignocellulosic biomass: biochemical and molecular perspectives. J Ind Microbiol Biotechnol.

[3] Singh A, Mishra P. Extraction of pentosans from lignocellulosic materials. In: Microbial pentose utilization—current applications in biotechnology. vol. 33; 1995. p. 71–98.

[4] Aro NPT, Penttilä M (2005). Transcriptional regulation of plant cell wall degradation by filamentous fungi. FEMS Microbiol Rev.

[5] Ivanova C, Bââth JA, Seiboth B, Kubicek CP (2013). Systems analysis of lactose metabolism in *Trichoderma reesei* identifies a lactose permease that is essential for cellulase induction. PLoS ONE.

[6] Mach-Aigner AR, Pucher ME, Mach RL (2010). D-Xylose as a repressor or inducer of xylanase expression in *Hypocrea jecorina* (*Trichoderma reesei*). Appl Environ Microbiol.

[7] Strauss J, Mach RL, Zeilinger S, Hartler G, Stoffler G, Wolschek M (1995). Cre1, the carbon catabolite repressor protein from *Trichoderma reesei*. FEBS Lett.

[8] Nakari-Setälä T, Paloheimo M, Kallio J, Vehmaanperä J, Penttilä M, Saloheimo M (2009). Genetic modification of carbon catabolite repression in *Trichoderma reesei* for improved protein production. Appl Environ Microbiol.

[9] Mach RL, Strauss J, Zeilinger S, Schindler M, Kubicek CP (1996). Carbon catabolite repression of xylanase I (*xyn1*) gene expression in *Trichoderma reesei*. Mol Microbiol.

[10] Portnoy T, Margeot A, Linke R, Atanasova L, Fekete E, Sandor E (2011). The CRE1 carbon catabolite repressor of the fungus *Trichoderma reesei*: a master regulator of carbon assimilation. BMC Genomics.

[11] Ries L, Belshaw NJ, Ilmén M, Penttilä ME, Alapuranen M, Archer DB (2014). The role of CRE1 in nucleosome positioning within the *cbh1* promoter and coding regions of *Trichoderma reesei*. Appl Microbiol Biotechnol.

[12] Stricker AR, Grosstessner-Hain K, Würleitner E, Mach RL (2006). Xyr1 (xylanase regulator 1) regulates both the hydrolytic enzyme system and D-xylose metabolism in *Hypocrea jecorina*. Eukaryot Cell.

[13] Mach-Aigner AR, Pucher ME, Steiger MG, Bauer GE, Preis SJ, Mach RL (2008). Transcriptional regulation of *xyr1*, encoding the main regulator of the xylanolytic and cellulolytic enzyme system in *Hypocrea jecorina*. Appl Environ Microbiol.

[14] Montenecourt BS, Eveleigh DE (1979). Production and characteriation of high yielding cellulase mutants of *Trichoderma reesei*. TAPPI J.

[15] Ward M. Improving secreted enzyme production by *Trichoderma reesei*. In: 9th International workshop on *Trichoderma* and *Gliocladium*: 2006; Vienna.

[16] Ilmén M, Thrane C, Penttilä M (1996). The glucose repressor gene *cre1* of *Trichoderma*: isolation and expression of a full-length and a truncated mutant form. Mol Gen Genetics.

[17] Mello-de-Sousa TM, Gorsche R, Rassinger A, Pocas-Fonseca MJ, Mach RL, Mach-Aigner AR (2014). A truncated form of the Carbon catabolite repressor 1 increases cellulase production in *Trichoderma reesei*. Biotechnol Biofuels.

[18] Seiboth B, Gamauf C, Pail M, Hartl L, Kubicek CP (2007). The D-xylose reductase of *Hypocrea jecorina* is the major aldose reductase in pentose and D-galactose catabolism and necessary for beta-galactosidase and cellulase induction by lactose. Mol Microbiol.

[19] Stricker AR, Steiger MG, Mach RL (2007). Xyr1 receives the lactose induction signal and regulates lactose metabolism in *Hypocrea jecorina*. FEBS Lett.

[20] Cziferszky A, Mach RL, Kubicek CP (2002). Phosphorylation positively regulates DNA binding of the carbon catabolite repressor Cre1 of *Hypocrea jecorina* (*Trichoderma reesei*). J Biol Chem.

[21] Lichius A, Seidl-Seiboth V, Seiboth B, Kubicek CP (2014). Nucleo-cytoplasmic shuttling dynamics of the transcriptional regulators XYR1 and CRE1 under conditions of cellulase and xylanase gene expression in *Trichoderma reesei*. Mol Microbiol.

[22] Tilburn J, Sarkar S, Widdick DA, Espeso EA, Orejas M, Mungroo J (1995). The Aspergillus PacC zinc finger transcription factor mediates regulation of both acid- and alkaline-expressed genes by ambient pH. EMBO J.

[23] Cánovas D, Studt L, Marcos AT, Strauss J (2017). High-throughput format for the phenotyping of fungi on solid substrates. Sci Rep.

[24] Sun J, Glass LN (2011). Identification of the CRE-1 cellulotytic regulon in *Neurospora crassa*. PLoS ONE.

[25] dos Santos Castro L, Pedersoli WR, Antonieto AC, Steindorff AS, Silva-Rocha R, Martinez-Rossi NM (2014). Comparative metabolism of cellulose, sophorose and glucose in *Trichoderma reesei* using high-throughput genomic and proteomic analyses. Biotechnol Biofuels.

[26] Derntl C, Gudynaite-Savitch L, Calixte S, White T, Mach RL, Mach-Aigner AR (2013). Mutation of the Xylanase regulator 1 causes a glucose blind hydrolase expressing phenotype in industrially used *Trichoderma* strains. Biotechnol Biofuels.

[27] NetNES 1.1 Server. http://www.cbs.dtu.dk/services/NetNES/. Accessed 31 May 2017.

[28] Ries L, Beattie SR, Espeso EA, Cramer RA, Goldman GH (2016). Diverse Regulation of CreA Carbon Catabolite Repressor in *Aspergillus nidulans*. Genetics.

[29] Ries L, Belshaw NJ, Ilmén M, Penttilä ME, Alapuranen M, Archer DB (2014). The role of CRE1 in nucleosome positioning within the *cbh1* promoter and coding regions of *Trichoderma reesei*. Appl Microbiol Biotechnol.

[30] Zeilinger S, Schmoll M, Pail M, Mach RL, Kubicek CP (2003). Nucleosome transactions on the *Hypocrea jecorina* (*Trichoderma reesei*) cellulase promoter *cbh2* associated with cellulase induction. Mol Genet Genomics.

[31] Steiger MG, Vitikainen M, Uskonen P, Brunner K, Adam G, Pakula T (2011). Transformation system for *Hypocrea jecorina* (*Trichoderma reesei*) that favors homologous integration and employs reusable bidirectionally selectable markers. Appl Environ Microbiol.

[32] Mach RL, Schindler M, Kubicek CP (1994). Transformation of *Trichoderma reesei* based on hygromycin B resistance using homologous expression signals. Curr Genet.

[33] Derntl C, Kiesenhofer DP, Mach RL, Mach-Aigner AR (2015). Novel strategies for genomic manipulation of *Trichoderma reesei* with the purpose of strain engineering. Appl Environ Microbiol.

[34] Gruber F, Visser J, Kubicek CP, de Graaff LH (1990). Cloning of the *Trichoderma reesei pyrG* gene and its use as a homologous marker for a high-frequency transformation system. Curr Genet.

[35] Steiger MG, Mach RL, Mach-Aigner AR (2010). An accurate normalization strategy for RT-qPCR in *Hypocrea jecorina* (*Trichoderma reesei*). J Biotechnol.

[36] JGI *Trichoderma reesei* v2.0 Genome Database. http://genome.jgi.doe.gov/Trire2/Trire2.home.html. Accessed 28 April 2014.

[37] JGI *Trichoderma reesei* Rut C-30 v1.0 Genome Database. http://genome.jgi.doe.gov/TrireRUTC30_1/TrireRUTC30_1.home.html. Accessed 28 April 2014.

[38] NCBI Conserved Domain Search. http://www.ncbi.nlm.nih.gov/Structure/cdd/wrpsb.cgi. Accessed 01 June 2014.

[39] Clustal Omega. http://www.ebi.ac.uk/Tools/msa/clustalo/. Accessed 13 June 2017.

[40] cNLS Mapper. http://nls-mapper.iab.keio.ac.jp/cgi-bin/NLS_Mapper_form.cgi. Accessed 08 September 2014.

[41] Nine Amino Acids Transactivation Domain (9aaTAD) Prediction Tool. http://www.med.muni.cz/9aaTAD/. Accessed 25 May 2017.

[42] Piskacek S, Gregor M, Nemethova M, Grabner M, Kovarik P, Piskacek M (2007). Nine-amino-acid transactivation domain: establishment and prediction utilities. Genomics.

[43] Schindelin J, Arganda-Carreras I, Frise E, Kaynig V, Longair M, Pietzsch T (2012). Fiji: an open-source platform for biological-image analysis. Nat Methods.

[44] Lando D, Endesfelder U, Berger H, Subramanian L, Dunne PD, McColl J (2012). Quantitative single-molecule microscopy reveals that CENP-ACnp1 depostiton occurs during G2 in fission yeast. Open Biol.

[45] Mello-de-Sousa TM, Rassinger A, Derntl C, Poças-Fonseca MJ, Mach-Aigner AR, Mach RL (2016). The relation between chromatin status, Xyr1 and cellulase expression in *Trichoderma reesei*. Curr Genomics.

[46] Mello-de-Sousa TM, Rassinger A, Pucher ME, dos Santos Castro L, Persinoti GF, Silva-Rocha R (2015). The impact of chromatin remodelling on cellulase expression in *Trichoderma reesei*. BMC Genomics.

