# Supplementary material for: Truncation of the transcriptional repressor protein Cre1 in Trichoderma reesei Rut-C30 turns it into an activator
Source: Fungal Biol Biotechnol. 2018 Aug 20;5:15. doi: 10.1186/s40694-018-0059-0 (PMC6100732; doi:10.1186/s40694-018-0059-0)
Supplement: Supplementary file 1 — Additional file 1: Figure S1. Deletion of cre1-96 in T. reesei Rut-C30. (A) Rut-C30 was transformed with the plasmid pMS*-5hph3cre1 that bears the deletion cassette consisting of the hygromycin resistance gene under the pki promoter and the terminator of cbh2 (dark grey arrow, hph) to replace the native cre1-96 gene (light grey arrow, cre1-96). (B) Agarose gel electrophoresis of diagnostic PCR was performed. Primer pairs added to the respective PCR are indicated on top of the gel, the strain of which the genomic DNA was used as template is indicated below each lane. Candidate strains (Δcre1-96 (1) and (2)) yielded expected fragments with the primer pair 1F and 1R or 3F and 3R, and no fragment in case of primer pair 2F and 2R. Rut-C30 was applied as negative control in the case of the PCR using primer pair 1F and 1R and as positive control in the PCR using primer pair 2F and 2R. Water added to the respective PCR in a no template control PCR (NTC). A DNA ladder (L) was included for estimation of fragment size. [file 40694_2018_59_MOESM1_ESM.pdf]

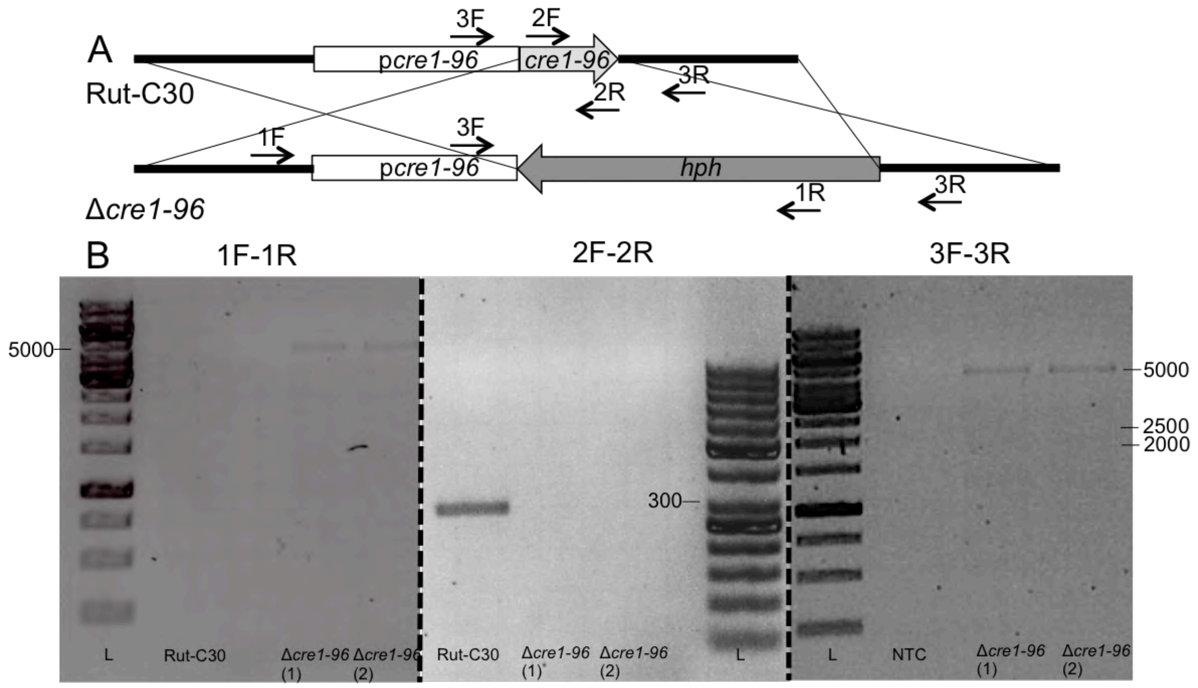

Figure S 1 – Deletion of *cre1-96* in *T. reesei* Rut-C30

(A) Rut-C30 was transformed with the plasmid pMS*-5hph3cre1 that bears the deletion cassette consisting of the hygromycin resistance gene under the *pki* promoter and the terminator of *cbh2* (dark grey arrow, *hph*) to replace the native *cre1-96* gene (light grey arrow, *cre1-96*). (B) Agarose gel electrophoresis of diagnostic PCR was performed. Primer pairs added to the respective PCR are indicated on top of the gel, the strain of which the genomic DNA was used as template is indicated below each lane. Candidate strains (Δ*cre1-96* (1) and (2)) yielded expected fragments with the primer pair 1F and 1R or 3F and 3R, and no fragment in case of primer pair 2F and 2R. Rut-C30 was applied as negative control in the case of the PCR using primer pair 1F and 1R and as positive control in the PCR using primer pair 2F and 2R. Water added to the respective PCR in a no template control PCR (NTC). A DNA ladder (L) was included for estimation of fragment size.
