# Supplementary material for: Truncation of the transcriptional repressor protein Cre1 in Trichoderma reesei Rut-C30 turns it into an activator
Source: Fungal Biol Biotechnol. 2018 Aug 20;5:15. doi: 10.1186/s40694-018-0059-0 (PMC6100732; doi:10.1186/s40694-018-0059-0)
Supplement: Supplementary file 2 — Additional file 2: Figure S2. Constitutive expression of cre1-96 in T. reesei Rut-C30. (A) Rut-C30 was transformed with the plasmid pMS*-ptef::cre1-96 that bears the tef1 promoter (white bar, ptef1), the cre1-96 gene (light grey arrow, cre1-96), and the marker cassette (dark grey bar, hph). The latter consists of the hygromycin resistance gene under the pki promoter and the terminator of cbh2. (B) Agarose gel electrophoresis of diagnostic PCR was performed. Primer pairs added to the respective PCR are indicated on top of the gel, the strain of which the genomic DNA was used as template is indicated below each lane. A candidate strain (OEcre1-96) yielded expected fragments with all three primer pairs. Rut-C30 was applied as negative control in case of the PCR using primer pair 1F and 1R as well as 2F and 2R and as a positive control in the PCR using primer pair 3F and 3R. A DNA ladder (L) was included for estimation of fragment size. [file 40694_2018_59_MOESM2_ESM.pdf]

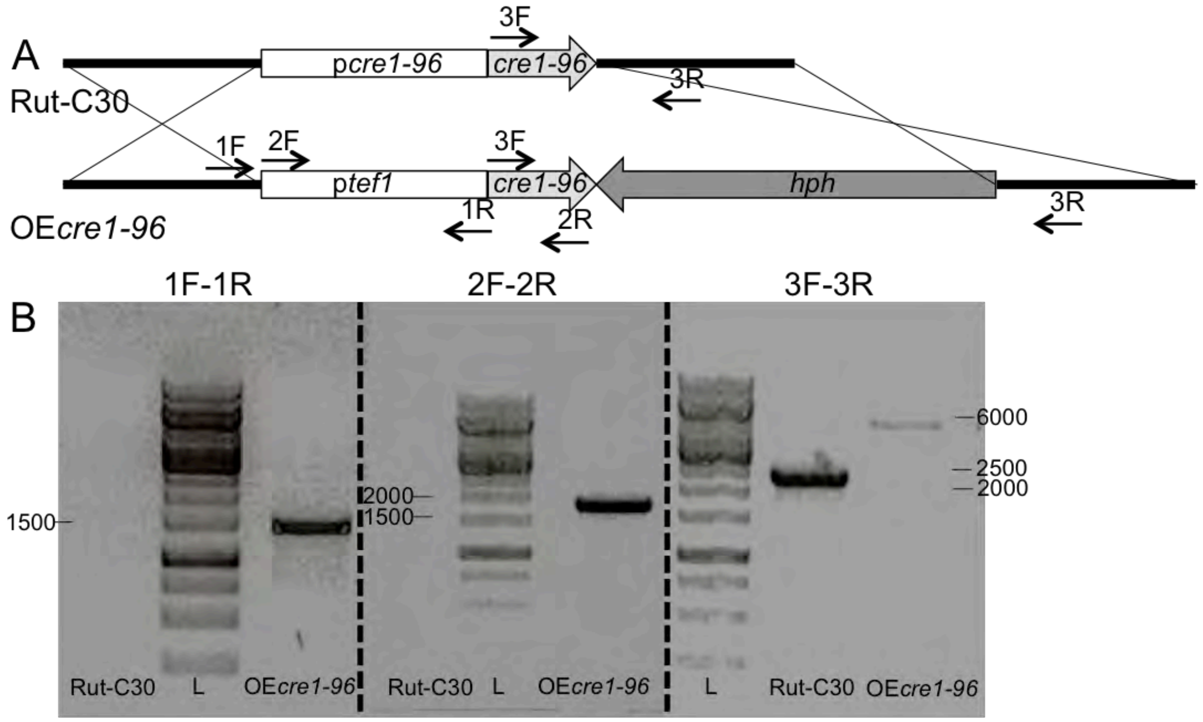

Figure S 2 – Constitutive expression of *cre1-96* in *T. reesei* Rut-C30

(A) Rut-C30 was transformed with the plasmid pMS*-*ptef*::*cre1-96* that bears the *tef1* promoter (white bar, p*tef1*), the *cre1-96* gene (light grey arrow, *cre1-96*), and the marker cassette (dark grey bar, *hph*). The latter consists of the hygromycin resistance gene under the *pki* promoter and the terminator of *cbh2*. (B) Agarose gel electrophoresis of diagnostic PCR was performed. Primer pairs added to the respective PCR are indicated on top of the gel, the strain of which the genomic DNA was used as template is indicated below each lane. A candidate strain (OE*cre1-96*) yielded expected fragments with all three primer pairs. Rut-C30 was applied as negative control in case of the PCR using primer pair 1F and 1R as well as 2F and 2R and as a positive control in the PCR using primer pair 3F and 3R. A DNA ladder (L) was included for estimation of fragment size.
