# Supplementary material for: Truncation of the transcriptional repressor protein Cre1 in Trichoderma reesei Rut-C30 turns it into an activator
Source: Fungal Biol Biotechnol. 2018 Aug 20;5:15. doi: 10.1186/s40694-018-0059-0 (PMC6100732; doi:10.1186/s40694-018-0059-0)
Supplement: Supplementary file 4 — Additional file 4: Figure S4. Multiple sequence alignment of Cre1 homologues. Multiple sequence alignment of T. reesei Cre1, Cre1-96 and Cre1 homologues of A. nidulans, A. niger, N. crassa, T. atroviride, T. virens and S. cerevisiae was conducted using Clustal Omega (http://www.ebi.ac.uk/Tools/msa/clustalo/). Protein sequences were retrieved from respective genome databases. The alignment revealed conserved amino acids and protein domains based on sequence similarities. [file 40694_2018_59_MOESM4_ESM.pdf]

CLUSTAL O(1.2.4) multiple sequence alignment

```
S. cerevisiae      MQSPYPMT------------------------------QVSNV-------DDGSLLKE--
A. nidulans        MPQPGSSVDFSNLLNPQNNTAIPA--------------EVSNATASATMASGASLLPPMV
A. niger           MPPPASSVDFSNLLNPQNNSTDSTPSTP--VDSSKTPSTPSSTQSNSNMASSVSLLPPLM
N. crassa          MQRVQSAVDFSNLLNPSESTAEKRDHSGSPRQQTAQPQQQQQQPQPEADMATVGLLRPNG
T. atroviride      MQRARSAVDFSNLLNPSSAAP---SQD---------------QGSGAMSTAAVTVIKPNG
T. virens          MQRAQSAVDFSNLLNPSSSAGQDSDAD---------------KGSGAMSTAAVTVIKPNG
T. reesei          MQRAQSAVDFSNLLNPTSAAGQD---------------------SGAMSTAAVTVIKPNG
  Cre1-96          MQRAQSAVDFSNLLNPTSAAGQD---------------------SGAMSTAAVTVIKPNG
                   *      .                                              ::

S. cerevisiae      -SKSKSKVAAKSEAPRPHACPICHRAFHRLEHQTRHMRIHTGEKPHACDFPGCVKRFSRS
A. nidulans        KGARPAAEEARQDLPRPYKCPLCERAFHRLEHQTRHIRTHTGEKPHACQFPGCSKRFSRS
A. niger           KGARPATEEVRQDLPRPYKCPLCDRAFHRLEHQTRHIRTHTGEKPHACQFPGCTKRFSRS
N. crassa          PLPGAQATEPANELPRPYKCPLCDKAFHRLEHQTRHIRTHTGEKPHACQFPGCSKKFSRS
T. atroviride      PIPGAQASESANELPRPYKCPLCDKAFRRLEHQTRHIRTHTGEKPHACQFPGCSKKFSRS
T. virens          PLPGAQSTETANELPRPYKCPLCEKAFHRLEHQTRHIRTHTGEKPHACQFPGCSKKFSRS
T. reesei          PIPGTQSTETANELPRPYKCPLCDKAFHRLEHQTRHIRTHTGEKPHACQFPGCSKKFSRS
  Cre1-96          PIPGTQSTETANELPRPYKCPLCDKAFHRLEHQTRHIRTHTGEKPHACTSITCFFF----
                              .: ***: **:*.:**:********:* *********    *

S. cerevisiae      DELTRHRRIHTNSHPRGKRGRKKKVVGSP-INSASS-SATSIPDLNTANFSPPLPQQHLS
A. nidulans        DELTRHSRIHNNPNSR--RGNKAQHLAAAAAAAAANQDGSAMANNAGSMMPPPSKPITRS
A. niger           DELTRHSRIHNNPNSR--RSNKAQHLAAAAAAAAG--QDNAMANTASAMMPPPSKPMTRS
N. crassa          DELTRHSRIHSNPNSR--RGNKGQQQQQHPLVHN--------HGLQPDMMPPPGPKAIRS
T. atroviride      DELTRHSRIHSNPNSR--RGNKGQQQHQQHL-HQGM---PHPLHVDGMMAPPPAPKAIRS
T. virens          DELTRHSRIHSNPNSR--RGNKGQQQHQQHLHHQGL---PHHMHVDGMMPP-PVPKAIRS
T. reesei          DELTRHSRIHSNPNSR--RGNKGQQQHQ--LHHQGM---PHPMHVDGLMHPPAAPKAIRS
  Cre1-96          ------------------------------------------------------------


S. cerevisiae      P----LIPIAIAPKEN-S-----SRSSTRKGRKTKFEIGESGGNDPYMVSSPKTMAKIPV
A. nidulans        APVSQVGSPDISPPHSFSNYANHMRSNLSPYS--RTSERASSGMDINLLATAASQVERDE
A. niger           APVSQVGSPDISPPHSFSNYASHMRSNLGPYA--RKGERASSGMDINLLATAASQVERDE
N. crassa          APPTAMSSPNVSPPHSYSPYNFAP-SGLNPYSHSRSSAGSQSGPDISLLARAAGQVERDG
```

```
T. atroviride     APASALVSPNVSPPHSYSSFAVPA-VSMPHYG---------RGTDISMLANAAHQIERET
T. virens         APTSTLVSPNVSPPHSYSSFVMPQ-APMPHYN---------RGNDITMLAKAANQIERET
T. reesei         APPSTLVSPNVSPPHSYSSFVMPH-GPISHYG---------RGNDITMLAKAANQIERET
  Cre1-96         ------------------------------------------------------------

S. cerevisiae     SVKPPPSLALNNMNYQTSSASTALSSLSNSHSGSRLKLNALSSLQMMTPIASSAPRTVFI
A. nidulans       SFGFR--SGQRSHHMYGPR---------H----GSRGLPSLSAYAISHSMSR----SHSH
A. niger          HFSFH--AGPRNHHLFSSR--------HH----GSGRLPSLSAYAITHNMSR----SHSH
N. crassa         AA---HHHFQPRFQFYGNT--------LHAATASRNQLPGLQAYH----MSR----SHSH
T. atroviride     LSGGPSNHNSRHHPYFSPG--------M---QGPRGHGPSLSSYH----MAR----SHFN
T. virens         LSGGPSNHNSRHHPYFGQS--------M---QNSRGHPPSLSSYH----MAR----SHSN
T. reesei         LSGGPSNHNSRHHPYFGQG--------V---PGSRGHP-SLSSYH----MAR----AHSN
  Cre1-96         ------------------------------------------------------------

S. cerevisiae     DGPEQK-------Q------LQQQQNSLSPRYSNTVILPRPRSLTDFQGLNNANPNNNGS
A. nidulans       EDED-SY--ASHRVKRSRPNSPNSTAPSSPTFSHDSLSPTPDHTP------LATPAHSPR
A. niger          EDDD-GY---SHRVKRSRPNSPNSTAPSSPTFSHDSLSPTPDHTP------LATPAHSPR
N. crassa         EDHDDHYGQSYRHAKRSRPNSPNSTAPSSPTFSHDSLSPTPDHTP------LATPAHSPR
T. atroviride     D-DDDHYHGSMRHAKRSRPNSPNSTAPSSPTFSHDSLSPTPDHTP------IATPAHSPR
T. virens         D-DDDHYS-SMRHAKRSRPNSPNSTAPSSPTFSHDSLSPTPDHTP------IATPAHSPR
T. reesei         D-EDDHYHGSLRHAKRSRPNSPNSTAPSSPTFSHDSLSPTPDHTP------IATPAHSPR
  Cre1-96         ------------------------------------------------------------

S. cerevisiae     LRAQTQSSVQLKRPSSVLSLNDLLVGQRNTNES---------------------------
A. nidulans       LKPLSPSELH------LPSIRHLSLH---HTPALAPMEPQAEGPNYYNPNQP----HVGP
A. niger          LRPLGSSDLH------LPSIRHLSLH---HTPALAPMEPQPEGPNYYSPSQG----HHGP
N. crassa         LRPHPG--LE------LPPFRNLSLGQQHTTPALAPLEPALDGQFSLPQTPPAAPRSSGM
T. atroviride     LRPFSG--YE------LPSLRNLSLGH-NTTPALAPMEPTLDTHQFPPQAQGLTSRGSGI
T. virens         LRPFSG--YE------LPSLRNLSLQH-NTTPALAPMEPHLDAPQFPPQLQANNNRSPGM
T. reesei         LRPFSG--YE------LPSLRNLSLQH-NTTPALAPMEPHLDAPQFHPQLQANTTRSPGM
  Cre1-96         ------------------------------------------------------------

S. cerevisiae     -DSDFTTGGEDEEDGLKDPSNSSIDNLEQDYLQEQSRKK-SKTSTPTTMLSRSTSGTNLH
```

```
A. nidulans       SISDIMSRPEGAQRKLPIPQV--PKVAVQDMLNP-SGFT-SVSSS----TANSVAGG---
A. niger          SISDIMSKPDGTQRKLPVPQV--PKVAVQDMLNPGSGFS-SVTSS----TANSVAGG---
N. crassa         SLTDIISRPDGTQRKLPVPK-----VAVQDLLGPADGFNPSVRNS----SSTSLSGA---
T. atroviride     SLTDIISRPDGSQRKLPVPQV--PKVAVQDLLSD-GIFTNSGRSS----TTGSLAGG---
T. virens         SLTDIISRPDGSQRKLPVPQV--PKVAVQDLLSD-GVFPNSGRSS----TAGSLAGG---
T. reesei         SLTDIISRPDGSQRKLPVPQV--PKVAVQDLLSD-GVFPNSGRSS----TTGSLAGG---
  Cre1-96         ------------------------------------------------------------

S. cerevisiae     TLGYVMNQNHLHFSSSSPDFQKELNNRLLNVQQQQQEQHTLLQSQNTSNQSQNQNQNQMM
A. nidulans       ----------------------DLAERF--------------------------------
A. niger          ----------------------DLAERF--------------------------------
N. crassa         ----------------------EMMDRL--------------------------------
T. atroviride     ----------------------DLMDRM--------------------------------
T. virens         ----------------------DLMDRM--------------------------------
T. reesei         ----------------------DLMDRM--------------------------------
  Cre1-96         ------------------------------------------------------------

S. cerevisiae     ASSSSLSTTPLLLSPRVNMINTAISTQQTPISQSDSQVQELETLPPIRSLPLPFPHMD
A. nidulans       ----------------------------------------------------------
A. niger          ----------------------------------------------------------
N. crassa         ----------------------------------------------------------
T. atroviride     ----------------------------------------------------------
T. virens         ----------------------------------------------------------
T. reesei         ----------------------------------------------------------
  Cre1-96         ----------------------------------------------------------
```

Figure S 4 – Multiple sequence alignment of Cre1 homologues

Multiple sequence alignment of *T. reesei* Cre1, Cre1-96 and Cre1 homologues of *A. nidulans*, *A. niger*, *N. crassa*, *T. atroviride*, *T. virens* and *S. cerevisiae* was conducted using Clustal Omega (http://www.ebi.ac.uk/Tools/msa/clustalo/). Protein sequences were retrieved from respective genome databases. The alignment revealed conserved amino acids and protein domains based on sequence similarities.
