# Supplementary material for: Truncation of the transcriptional repressor protein Cre1 in Trichoderma reesei Rut-C30 turns it into an activator
Source: Fungal Biol Biotechnol. 2018 Aug 20;5:15. doi: 10.1186/s40694-018-0059-0 (PMC6100732; doi:10.1186/s40694-018-0059-0)
Supplement: Supplementary file 5 — Additional file 5: Figure S5. Cellulase activity and biomass formation of QM6acre1-96 and QM6acre1-96::eyfp on d-glucose. The T. reesei strains QM6acre1-96 (blue bar, Cre1-96) and QM6acre1-96::eyfp (purple bar, Cre1-96::eYFP) were cultivated in triplicates for 45 hours in MA medium supplemented with 1 % (w/v) d-glucose. Cellulase activities (A) of the culture supernatants were measured in technical duplicates and the biomass (B) was collected by filtration with miracloth and is depicted as dry weight on the y-axis. [file 40694_2018_59_MOESM5_ESM.pdf]

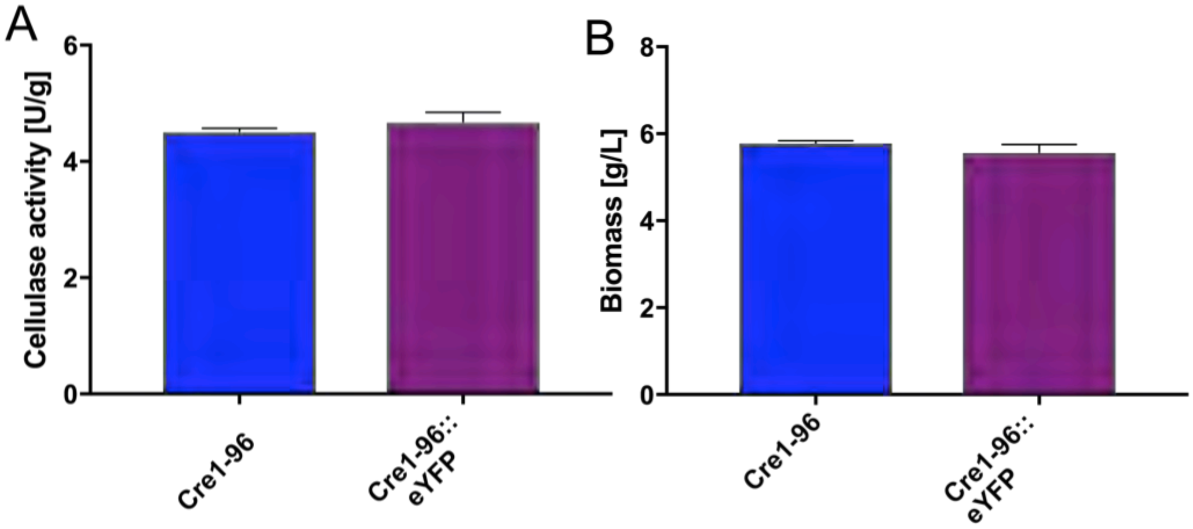

Figure S 5 – Cellulase activity and biomass formation of QM6a*cre1-96* and QM6a*cre1-96::eyfp* on D-glucose

The *T. reesei* strains QM6a*cre1-96* (blue bar, Cre1-96) and QM6a*cre1-96::eyfp* (purple bar, Cre1-96::eYFP) were cultivated in triplicates for 45 hours in MA medium supplemented with 1 % (w/v) D-glucose. Cellulase activities (A) of the culture supernatants were measured in technical duplicates and the biomass (B) was collected by filtration with miracloth and is depicted as dry weight on the y-axis.
