# Supplementary material for: Truncation of the transcriptional repressor protein Cre1 in Trichoderma reesei Rut-C30 turns it into an activator
Source: Fungal Biol Biotechnol. 2018 Aug 20;5:15. doi: 10.1186/s40694-018-0059-0 (PMC6100732; doi:10.1186/s40694-018-0059-0)
Supplement: Supplementary file 6 — Additional file 6: Figure S6. Growth behaviour of Rut-C30OEcre1-96 on glycerol and d-glucose. The T. reesei strains Rut-C30, Rut-C30Δcre1-96 (2) and Rut-C30OEcre1-96 were pre-grown on MEX plates and were then transferred to MA medium plates supplemented with 1 % (w/v) glycerol and d-glucose. Plates were incubated at 30 °C and pictures were taken after 24, 48, 60 and 84 hours. [file 40694_2018_59_MOESM6_ESM.pdf]

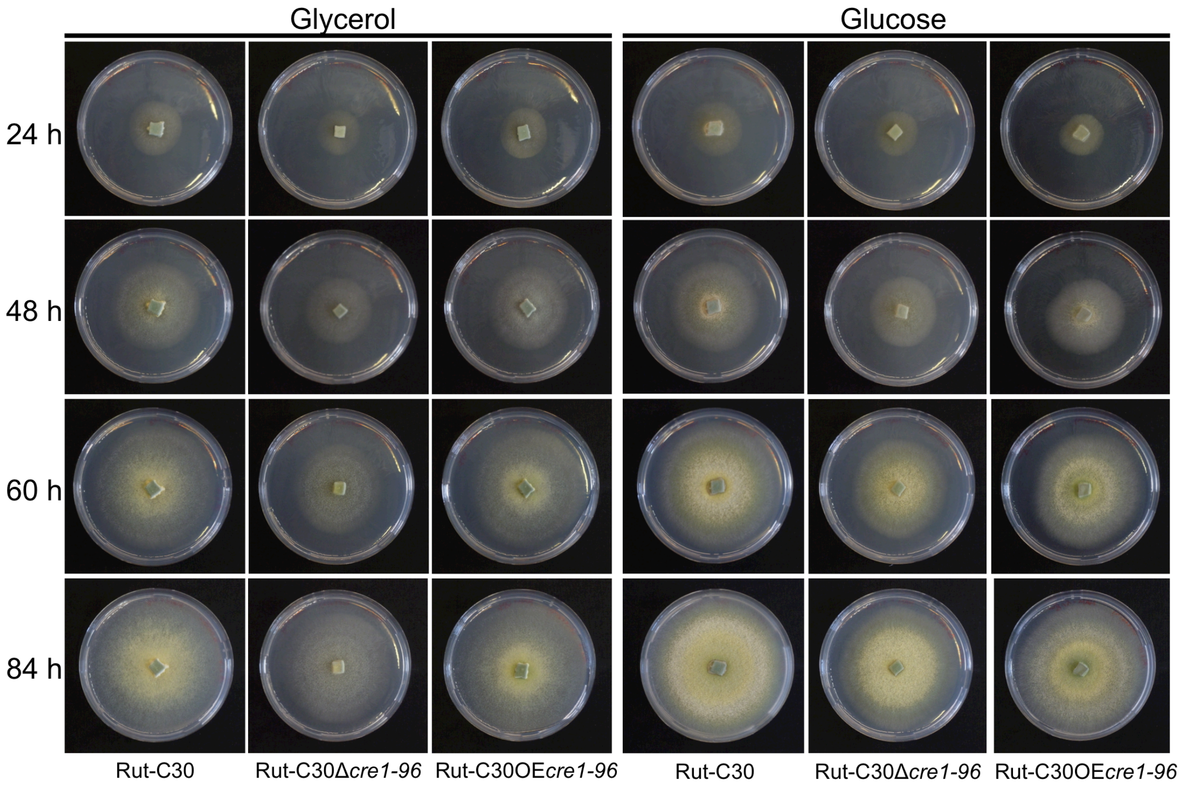

Figure S 6 – Growth behaviour of Rut-C30OE*cre1-96* on glycerol and D-glucose

The *T. reesei* strains Rut-C30, Rut-C30Δ*cre1-96* (2) and Rut-C30OE*cre1-96* were pre-grown on MEX plates and were then transferred to MA medium plates supplemented with 1 % (w/v) glycerol and D-glucose. Plates were incubated at 30 °C and pictures were taken after 24, 48, 60 and 84 hours.
