# Supplementary material for: Truncation of the transcriptional repressor protein Cre1 in Trichoderma reesei Rut-C30 turns it into an activator
Source: Fungal Biol Biotechnol. 2018 Aug 20;5:15. doi: 10.1186/s40694-018-0059-0 (PMC6100732; doi:10.1186/s40694-018-0059-0)
Supplement: Supplementary file 7 — Additional file 7: Table S1. Primers used for the diagnostic PCR of Rut-C30Δcre1-96 (1) and (2). [file 40694_2018_59_MOESM7_ESM.docx]

### Table S1 Primers used for the diagnostic PCR of Rut-C30Δ*cre1-96* (1) and (2).

| Abbreviation | Full primer name | Sequence 5’–3’ |
| --- | --- | --- |
| 1F | OE cre196 5'flank fwd | TCCGTCTCCAAGTTAGGTACTCC |
| 1R | RG201 | TCTAGCCTGATTCCAAGGTTGACC |
| 2F | cre1-96_BspEI fwd | TCCGGAATGCAACGAGCACAGTCTGCC |
| 2R | cre1-96_NdeI rev | CATATGTTAGAAAAAAAAGCAGGTAATGGAGGTGCAG |
| 3F | RG200 | CTTCTCTGGGCTCTCTTGTAAC |
| 3R | 3' flank cre1 locus rev | AGGATCCTTCTGCGGCTCAGC |
