# Supplementary material for: Truncation of the transcriptional repressor protein Cre1 in Trichoderma reesei Rut-C30 turns it into an activator
Source: Fungal Biol Biotechnol. 2018 Aug 20;5:15. doi: 10.1186/s40694-018-0059-0 (PMC6100732; doi:10.1186/s40694-018-0059-0)
Supplement: Supplementary file 8 — Additional file 8: Table S2. Primers used for the diagnostic PCR of Rut-C30OEcre1-96. [file 40694_2018_59_MOESM8_ESM.docx]

**Table S2** Primers used for the diagnostic PCR of Rut-C30OE*cre1-96*.

| Abbreviation | Full primer name | Sequence 5’–3’ |
| --- | --- | --- |
| 1F | OE cre196 5'flank fwd | TCCGTCTCCAAGTTAGGTACTCC |
| 1R | ptef_NdeI rev | CATATGTGACGGTTTGTGTGATGTAGCGTG |
| 2F | ptef_BspEI fwd | TCCGGATGTGTGACAGCTCGCGCAG |
| 2R | Cre1-96_BamHI rev | GGATCCTTAGAAAAAAAAGCAGGTAATGGAGGTGC |
| 3F | cre1-96_NdeI fwd | CATATGATGCAACGAGCACAGTCTGCC |
| 3R | 3' flank cre1 locus rev | AGGATCCTTCTGCGGCTCAGC |
